# Aurora-B phosphorylates the myosin II heavy chain to promote cytokinesis

**DOI:** 10.1016/j.jbc.2021.101024

**Published:** 2021-07-31

**Authors:** Aryeh Babkoff, Einav Cohen-Kfir, Hananel Aharon, Shoshana Ravid

**Affiliations:** Department of Biochemistry and Molecular Biology, The Institute of Medical Research Israel-Canada, The Hebrew University-Hadassah Medical School, Jerusalem, Israel

**Keywords:** myosin II, Aurora-B, survivin, cytokinesis, ACD, assembly competence domain, AZD, Aurora-B-specific inhibitor AZD1152-HQPA, cACD, complementary ACD, CPC, chromosomal passenger complex, INCENP, inner centromeric protein, L.PEI, linear PEI, MKLP2, mitotic kinesin-like protein 2, NMII, nonmuscle myosin II, PCC, Pearson’s correlation coefficient, PDL, Poly-DL-Lysine, STLC, S-trityl-L-cysteine

## Abstract

Cytokinesis, the final step of mitosis, is mediated by an actomyosin contractile ring, the formation of which is temporally and spatially regulated following anaphase onset. Aurora-B is a member of the chromosomal passenger complex, which regulates various processes during mitosis; it is not understood, however, how Aurora-B is involved in cytokinesis. Here, we show that Aurora-B and myosin-IIB form a complex *in vivo* during telophase. Aurora-B phosphorylates the myosin-IIB rod domain at threonine 1847 (T^1847^), abrogating the ability of myosin-IIB monomers to form filaments. Furthermore, phosphorylation of myosin-IIB filaments by Aurora-B also promotes filament disassembly. We show that myosin-IIB possessing a phosphomimetic mutation at T^1847^ was unable to rescue cytokinesis failure caused by myosin-IIB depletion. Cells expressing a phosphoresistant mutation at T^1847^ had significantly longer intercellular bridges, implying that Aurora-B-mediated phosphorylation of myosin-IIB is important for abscission. We propose that myosin-IIB is a substrate of Aurora-B and reveal a new mechanism of myosin-IIB regulation by Aurora-B in the late stages of mitosis.

Cytokinesis, the last step of cell division, is a highly regulated process that involves many components. Defects in this process can result in aneuploidy, a feature that has been related to chromosomal instability and carcinogenesis (1, 2). One of the requirements for proper cytokinesis is the coordination between chromosome segregation and the separation of the cytoplasmic content. To achieve this, the cell must control the assembly and constriction of an equatorial contractile ring composed of F-actin, nonmuscle myosin II (NMII), and other cytoskeletal components (3). Numerous studies have suggested that dysregulation of contractile ring formation leads to mitotic defects (4, 5, 6).

In vertebrates, there are at least three NMII isoforms: NMIIA, NMIIB, and NMIIC (7, 8, 9). The NMII molecule is composed of two heavy chains and two pairs of light chains (MLC). The heavy chains consist of an N-terminal motor domain containing the actin-binding and ATPase activity and a tail domain (10). The tail domain is responsible for the assembly of NMII monomers into filaments, which are the functional structures required for NMII activity (11, 12, 13, 14). Studies in multiple systems indicate that, in addition to filament formation, the tail of NMII holds targeting signals to different compartments, including the cleavage-furrow (13, 15, 16, 17, 18). NMII functions are regulated within the cell in several ways. An example of this is the regulation through protein–protein interaction. Many studies have shown that different proteins can bind directly to NMII, regulating its filament assembly (11, 19, 20, 21). Another regulatory mechanism operates through the phosphorylation of NMII heavy and light chains (10). Several studies using mammalian cells have shown that NMII heavy chains can be phosphorylated by different kinases, such as protein kinase C (PKC) and casein kinase II (11, 22, 23, 24). These phosphorylation events have been shown to regulate NMII functions, such as cellular localization, protein–protein interactions, and filament assembly (12, 24, 25, 26). For example, phosphorylation of NMIIB heavy chains by PKCγ regulates its filament assembly and cellular localization (24). It has also been shown that NMIIB phosphorylation is important for the regulation of front–back cell polarity (26). *Dictyostelium* NMII heavy chain phosphorylation has been shown to play important role in cytokinesis (27, 28). Thus, phosphorylation of NMII heavy chains is associated with the regulation of directed cell migration and cytokinesis (29, 30, 31, 32).

The chromosomal passenger complex (CPC) is a protein complex composed of Aurora-B kinase, inner centromeric protein (INCENP), borealin, and survivin (33). The CPC contains two modules connected by INECNP: the localization module, composed of survivin, borealin, and the N terminus of INCENP, and the kinase module, composed of the C terminus of INCENP and the Aurora-B kinase (34, 35). The CPC plays several roles throughout mitosis, including cytokinesis and abscission (33). This is achieved by dynamic localization changes through mitosis (36). Perturbation in the function of any of the CPC components results in mislocalization of the other components as well (33). Several studies have shown that the CPC plays an important role in regulating the contractile ring (37, 38, 39). For instance, it was suggested that the *Caenorhabditis elegans* Aurora-B promotes RhoA activation through centralspindlin, which may lead to NMII activation (37). In mammals, however, it has been shown that the RhoA cortical region during mitosis was expanded upon Aurora-B inhibition (39). In another study, it was shown that the cleavage furrow only partially ingresses and at times even regressed upon Aurora-B inhibition (38). Finally, we recently showed that survivin regulates the spatiotemporal formation of the acto-NMII contractile ring during cytokinesis (19).

Aurora-B is a member of the Aurora serine/threonine protein kinases. In mammals, there are three kinases in the Aurora kinase family: A, B, and C (40). Aurora-B overexpression has been associated with aneuploidy and with poor survival in oncology patients (41, 42, 43). Consistent with this, several studies have shown that Aurora-B regulates numerous processes throughout mitosis, including spindle assembly checkpoint activation, membrane ingression during telophase, and abscission (44, 45, 46, 47, 48). Many of the processes described are regulated through phosphorylation of Aurora-B substrates (49, 50). Indeed, it has been shown that, during mitosis, there is a gradient of phosphorylated Aurora-B substrates that provides spatial information for positioning the cleavage furrow (51). Moreover, several studies have shown that aberrant regulation of Aurora-B activity causes mitotic defects (52). Hyperactivated Aurora-B caused mitotic defects, but rescue experiments with phosphomimetic Aurora-B substrates were not able to fully rescue mitotic defects caused by the depletion of the endogenous protein (53, 54). Thus, Aurora-B may phosphorylate unidentified substrate(s) that are important for mitosis. It has been shown that Aurora-B phosphorylates the NMII light chains and that Aurora-B colocalized with and phosphorylated light chains at the cell cleavage furrow of dividing cells (55). Thus, Aurora-B may participate in phosphorylation of NMII light chains during cytokinesis (55).

Here, we report that Aurora-B forms a complex with NMIIB *in vivo* and that these proteins colocalize during late stages of mitosis. We identified the coiled-coil rod of NMIIB as a novel substrate of Aurora-B. The Aurora-B phosphorylation site at NMIIB (T^1847^) resides within a region important for filament assembly and binding to survivin. We show that Aurora-B phosphorylation modulates NMIIB filament structure and assembly properties *in vitro* and that Aurora-B phosphorylation of NMIIB leads to filament disassembly. Moreover, phosphomimetic NMIIB was unable to rescue multinucleation caused by NMIIB depletion in COS-7 cells, attesting to the importance of Aurora-B phosphorylation for the proper function of NMIIB in cytokinesis. Furthermore, NMIIB equatorial cortex localization during telophase is dependent on Aurora-B activity. Finally, we show that Aurora-B phosphorylation of NMIIB regulates NMIIB–survivin interaction. Thus, our results reveal the important role of Aurora-B in mitosis. To our knowledge, this is the first study demonstrating the role of NMII heavy chain phosphorylation by Aurora-B in the regulation of NMII during mitosis.

## Results

### Aurora-B and NMIIB form a complex *in vivo*

**Figure 1:**
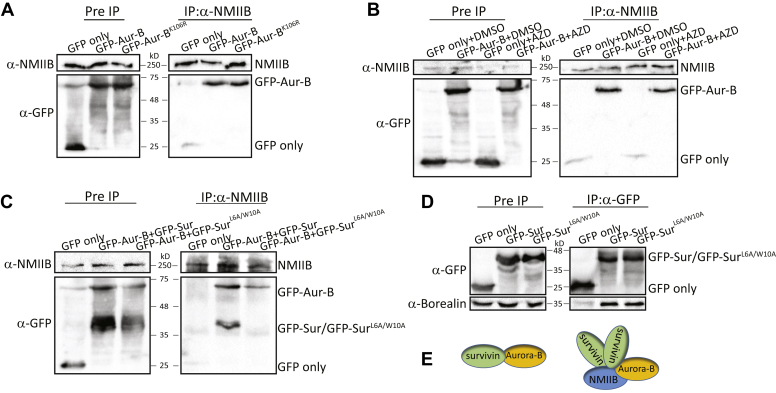
**Aurora-B and NMIIB form a complex *in vivo*.***A*, 293T cells were transfected with GFP-Aurora-B or GFP-Aurora-B^K106R^ and subjected to coimmunoprecipitation (co-IP) assay with endogenous NMIIB using anti-NMIIB antibody. The immunoprecipitated (IP) proteins were analyzed by immunoblotting (IB) with antibodies against NMIIB and GFP. GFP alone was used as a negative control. *B*, 293T cells expressing GFP only or GFP-Aurora-B were treated with Aurora-B inhibitor AZD for an hour and subjected to co-IP assay using anti-NMIIB antibody. The IP proteins were analyzed by IB with anti-NMIIB and anti-GFP antibodies. Beads only and IP in the presence of dimethyl sulfoxide served as negative and positive controls, respectively. *C*, 293T cells were cotransfected with GFP-Aurora-B and GFP-survivin or GFP-Aurora-B and GFP-survivin^L6A/W10A^ and subjected to co-IP assay with endogenous NMIIB using anti-NMIIB antibody. The IP proteins were analyzed by IB with antibodies against NMIIB and GFP. GFP alone was used as a negative control. *D*, 293T cells were transfected with GFP-survivin or GFP-survivin^F101A/L102A^ and subjected to co-IP assay using anti-GFP antibody. The IP proteins were analyzed by IB with antibodies against Borealin and GFP. GFP alone was used as a negative control. *E*, a model depicting the different complexes that are formed by NMIIB, survivin, and Aurora-B during mitosis.

Aurora-B and NMIIB are important for cytokinesis (56, 57). As survivin interacts with Aurora-B (58) and NMIIB (19) during mitosis, we hypothesized that Aurora-B and NMIIB form a complex *in vivo*. To test our hypothesis, we transfected 293T with GFP-Aurora-B and subjected it to coimmunoprecipitation assay using anti-NMIIB antibody. We found that GFP-Aurora-B coimmunoprecipitated with endogenous NMIIB (Fig. 1*A*), thus forming a complex *in vivo*. Next, we tested whether Aurora-B activity is required for its interaction with NMIIB. To this end, we tested whether Aurora-B with a dominant negative mutation (Aurora-B^K106R^) leading to inactive Aurora-B (59) coimmunoprecipitates with endogenous NMIIB. We found that inactive Aurora-B was capable of forming a complex with NMIIB *in vivo* (Fig. 1*A*). Furthermore, we found that Aurora-B and NMIIB form a complex *in vivo* in the presence of the Aurora-B-specific inhibitor AZD1152-HQPA (AZD) (60) (Fig. 1*B*). These results indicate that Aurora-B activity is not required for its interaction with NMIIB.

### Aurora-B forms a ternary complex with NMIIB and homodimer survivin

As Aurora-B forms a complex with survivin (58) and with NMIIB (19), we tested whether the three proteins reside in one complex. To this end, we cotransfected HEK293T cells with GFP-survivin and GFP-Aurora-B, and subjected them to coimmunoprecipitation assay with endogenous NMIIB, using anti-NMIIB antibody. Endogenous NMIIB coimmunoprecipitated with GFP-survivin and GFP-Aurora-B, but not with GFP, indicating the specificity of the interaction (Fig. 1*C*), confirming that the three proteins reside in a complex *in vivo*. We have shown previously that the NMIIB–survivin complex is formed only when survivin is in homodimers (19). Therefore, we tested whether survivin dimerization is essential for the formation of the ternary complex NMIIB–survivin–Aurora-B. To achieve this, we cotransfected HEK293T cells with GFP-Aurora-B and the dimerization mutant GFP-survivin^L6A/W10A^ (19) and subjected them to coimmunoprecipitation assay with endogenous NMIIB. GFP-survivin^L6A/W10A^ was unable to coimmunoprecipitate with NMIIB despite the fact that GFP-Aurora-B formed a complex with NMIIB (Fig. 1*C*). These results indicate that survivin dimerization is necessary to form the ternary complex NMIIB–survivin–Aurora-B. Note that the inability of GFP-survivin^L6A/W10A^ to form a complex with NMIIB is probably not a result of survivin inability to form a complex with Aurora-B, because it has been shown that survivin monomer is able to bind the CPC *in vitro* (19, 34). To further confirm that survivin monomer is able to form a complex with the CPC *in vivo*, we transfected 293T cells with GFP-survivin^L6A/W10A^ and subjected them to coimmunoprecipitation assay with endogenous Borealin, another member of the CPC complex (33, 34) using anti-GFP antibody. As shown in Figure 1*D*, GFP-survivin and GFP-survivin^L6/W10A^ coimmunoprecipitated with endogenous Borealin (Fig. 1*D*), indicating that survivin monomer forms a complex with the CPC *in vivo*. These results indicate that Aurora-B forms at least two discrete complexes, Aurora-B–survivin monomer, as part of the CPC and Aurora-B–NMIIB and survivin homodimer (Fig. 1*E*).

### Aurora-B and NMIIB colocalize during late stages of mitosis

**Figure 2:**
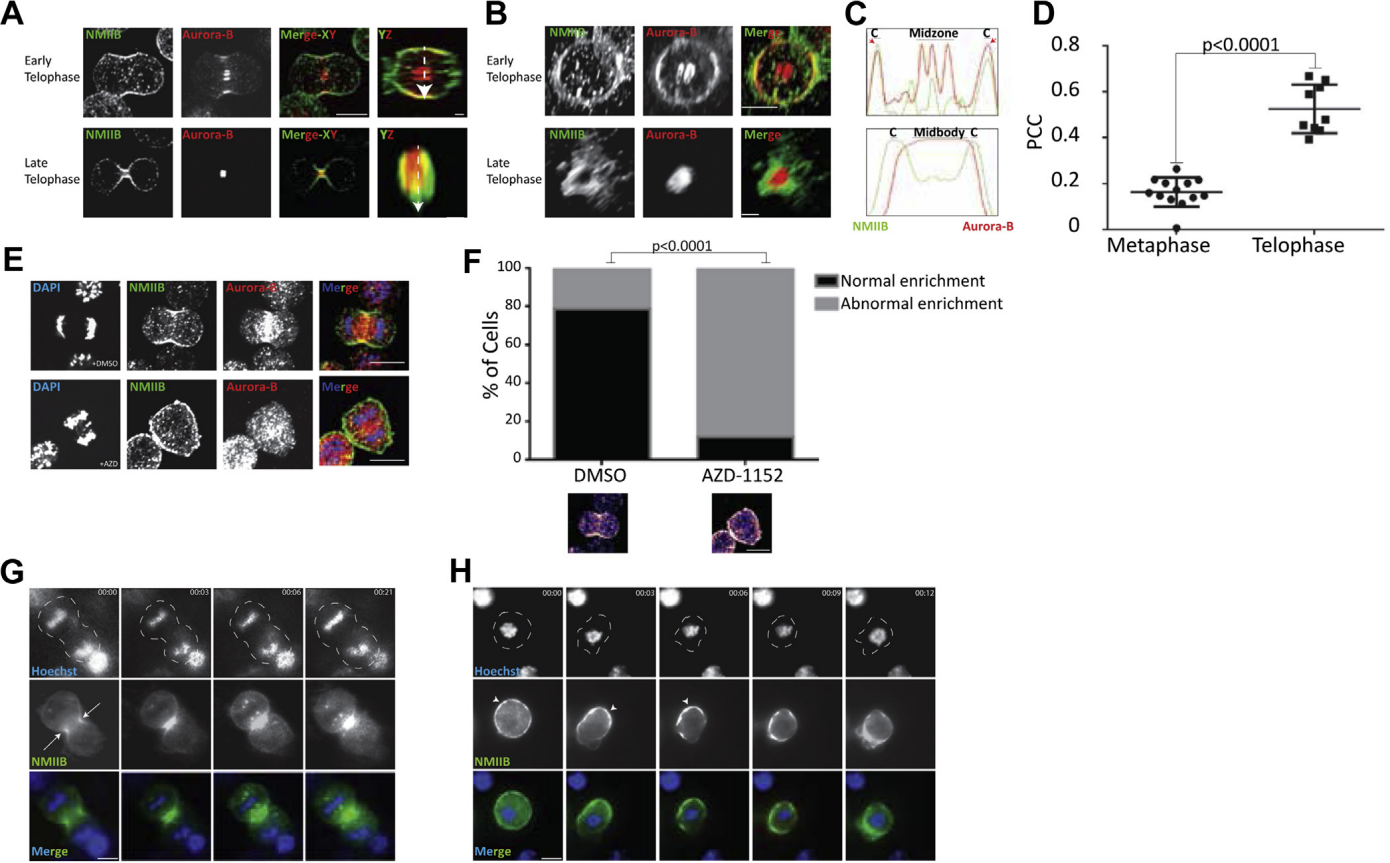
**Aurora-B and NMIIB colocalize during telophase.***A*, HeLa cells were synchronized, seeded on Poly-DL-Lysine–coated coverslips, fixed, and immunostained for endogenous NMIIB (*green*) and Aurora-B (*red*). Shown are single Z-plane micrographs (XY) and volume projections of NMIIB and Aurora-B in YZ dimensions throughout mitosis. Tubulin was used to determine the mitotic stage of each cell (not shown). The scale bar represents 10 μm in the XY plane and 2.5 μm in the YZ plane. Note that the doted staining of NMIIB represents cytoplasmic NMIIB. *B*, 3D reconstitution of NMIIB (*green*) and Aurora-B (*red*) localization in HeLa cells shown in *A*. Note that the disperse staining of NMIIB was originated from cytoplasmic NMIIB. *C*, normalized fluorescence intensity was measured along the *white dashed arrows* shown in *A*. Colocalization is indicated by the *red arrows*. *D*, Pearson correlation coefficient (PCC) between the intensity of the fluorescence of endogenous Aurora-B and NMIIB in the cell cortex of metaphase cells and in telophase cells, as described (19). Results are mean ± standard error of mean (SEM), n = 13 and 9, respectively. *E*, HeLa cells were synchronized and seeded on Poly-DL-Lysine–coated coverslips. An hour before fixation, cells were treated with dimethyl sulfoxide (DMSO) or AZD, as indicated. Next, cells were fixed and immunostained for endogenous NMIIB (*green*) and Aurora-B (*red*). *F, top*, quantification of HeLa cells treated with DMSO or AZD. Data are mean ± SEM from three independent experiments (>15 cells/experiment for cells treated with DMSO and >30 cells/experiment for cells treated with AZD). *p* < 0.0001 was calculated by chi-square test. *Bottom*, fire representation of endogenous NMIIB along the cortex of representative mitotic HeLa cells treated with DMSO or AZD. To determine normal cortical enrichment, line scan was drawn along the cell cortex as in *C*. Cells presenting two significant peaks of NMIIB were referred to as normal enriched cells. The left and right cells represent normal and abnormal NMIIB enrichment, respectively. *G*, single Z-plane micrographs taken from a time-lapse movie of HeLa cells transiently expressing GFP-NMIIB treated with DMSO (Movie S1). Chromosomes were stained with Hoechst 33342. Images were taken every 3 min. Dashed line represents the cell periphery. *Arrows* represent GFP-NMIIB enrichment. The scale bar represents 10 μm. *H*, single Z-plane micrographs taken from a time-lapse movie of HeLa cells transiently expressing GFP-NMIIB treated with AZD (Movie S2). Chromosomes were stained using Hoechst 33342. Images were taken every 3 min. *Arrow* heads represent GFP-NMIIB abnormal enrichment. *Dashed lines* represent the cell periphery. The scale bar represents 10 μm. c, cell cortex

To begin understanding the role of the Aurora-B–NMIIB complex in mitosis, we determined where and when the Aurora-B–NMIIB complex is formed. Given that the survivin–NMIIB complex is a mitotic complex, we hypothesized that so is the Aurora-B–NMIIB complex. To test this hypothesis, we immunostained fixed HeLa cells for endogenous Aurora-B and NMIIB in different stages of mitosis. In early telophase, Aurora-B was located at the spindle midzone and appeared at the cell cortex colocalized with NMIIB (Fig. 2*A*). Most of NMIIB at this stage was cortical, forming a ring-like structure as viewed in volume projection (YZ dimensions) and 3D reconstruction (Fig. 2, *A* and *B*). In late telophase, Aurora-B localized mainly at the midbody, but some Aurora-B also localized at the equatorial cortex. NMIIB was located mainly at the cleavage plane, forming the contractile ring (Fig. 2, *A* and *B*). Line scan analysis indicated the Aurora-B and NMIIB colocalized mainly at the equatorial cortex in both phases of telophase (Fig. 2*C*). To further demonstrate that Aurora-B and NMIIB localize mainly during telophase, we calculated the Pearson’s correlation coefficient (PCC) between the fluorescence intensity profiles of NMIIB and Aurora-B in the equatorial cortex. Quantitatively, the PCC between NMIIB and Aurora-B in telophase (0.53 ± 0.04) was significantly higher than in metaphase (0.16 ± 0.02) (Figs. 2*D* and S1*A*). Thus, Aurora-B and NMIIB colocalize mainly in telophase.

To determine the timing of Aurora-B–NMIIB interaction, we tracked the localization of GFP-Aurora-B and mCherry–NMIIB through cell division in live HeLa cells. In early telophase, mCherry–NMIIB localized mainly in the equatorial cortex, and some of it created a diffuse band in the cell midzone (Fig. S1*B*; Movie S1). GFP-Aurora-B was mainly diffused, but it was enriched in the midzone and to some extent in the equatorial cortex (Fig. S1*B*; Movie S1). As mitosis progressed, GFP-Aurora-B concentrated in the cell midbody and mCherry–NMIIB at the midbody cortex (Fig. S1*B*; Movie S1). Thus, the immunofluorescence and live imaging results indicate that in telophase Aurora-B and NMIIB colocalized mainly at midbody cortex. Note that GFP-Aurora-B and mCherry–Aurora-B, as well as mCherry–NMIIB and GFP-NMIIB, showed similar localization patterns, indicating that the fluorescence protein tag did not affect the localization pattern of Aurora-B or NMIIB (data not shown). Moreover, GFP-Aurora-B and mCherry–NMIIB show a localization pattern similar to that of endogenous proteins, indicating that the expressed proteins mimic the characteristic localization profile of endogenous ones (Figs. 2*A* and S1*B*).

### NMIIB equatorial cortex localization depends on Aurora-B activity

Because Aurora-B activity is essential for cytokinesis (60), we hypothesized that its activity may affect the cellular localization of NMIIB. To test this hypothesis, we treated HeLa cells with AZD and immunostained them for endogenous Aurora-B and NMIIB. Aurora-B inhibition led to abnormal NMIIB cortical localization during early telophase (Fig. 2*E*). In addition, under AZD treatment, Aurora-B was diffused throughout the cell and showed some localization at the central spindle (Fig. 2*E*). Next, we quantified the percentage of cells with normal NMIIB enrichment at the cell cortex during telophase in the presence of AZD. Approximately 80% of control cells showed normal NMIIB cortical enrichment, whereas only 10% of cells treated with AZD did so (Fig. 2*F*). These results indicate that Aurora-B activity and localization are important for proper NMIIB localization during telophase.

Next, we tracked the localization of GFP-NMIIB in live HeLa cells treated with AZD. Control cells presented proper mitosis with GFP-NMIIB localization at the cleavage furrow (Fig. 2*G* and Movie S2). By contrast, cells treated with AZD showed mitotic defects, with NMIIB having aberrant cortical enrichment even before chromatids separation (Fig. 2*H* and Movie S3, Fig. S2 and Movie S4). The aberrant cellular localization of NMIIB during mitosis is not the result of disruption of the NMIIB–Aurora-B complex formation, because GFP-Aurora-B was able to form a complex with NMIIB in the presence of AZD (Fig. 1*B*). In addition, inactive Aurora-B (Aurora-B^K106R^) (59) coimmunoprecipitated with endogenous NMIIB (Fig. 1*A*). It was recently shown that Aurora-B inhibition does not affect RhoA and anillin localization (61), further indicating that aberrant NMIIB localization is not due to the absence of NMIIB–Aurora-B interactions.

Together, these results indicate that Aurora-B activity, and not its binding to NMIIB *per se*, is essential for the spatiotemporal regulation of the actomyosin contractile ring.

### Aurora-B phosphorylates the tail domain of NMIIB

**Figure 3:**
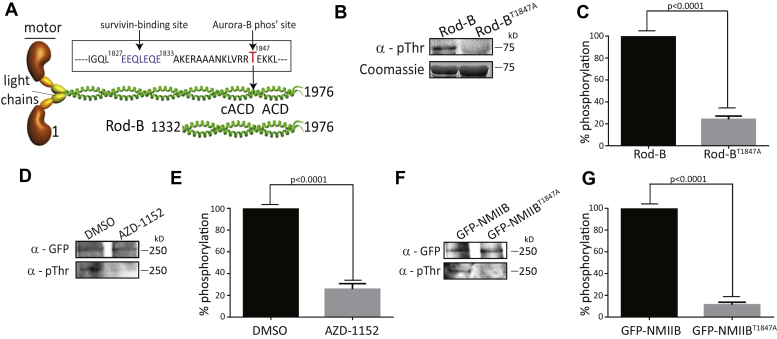
**Aurora-B phosphorylates NMIIB on threonine 1847 *in vivo* and *in vitro*.***A*, schematic presentation of NMIIB full length and Rod-B fragment. Amino acids 1827 to 1833 (marked in *blue*) are the survivin-binding domain in NMIIB (19). T^1847^ is the Aurora-B phosphorylation site (marked in *red*). This site is in close proximity to the survivin-binding domain. ACD and cACD are assembly competent domain and complementary ACD, respectively. Rod-B represents the protein used for the phosphorylation assay. *B*, purified Rod-B or Rod-B^T1847A^ was incubated with His-Aurora B and subjected to phosphorylation assay. Reactions were terminated with sample buffer. Proteins were loaded on SDS-PAGE and analyzed by IB using antibodies against Phospho-Threonine. *C*, densitometry analysis of Rod-B phosphoproteins presented in *B*. Phospho-Threonine signal was normalized to the total amount of the protein loaded (Coomassie). Error bars show the SEM from three independent experiments. *D*, HEK293T cells were cotransfected with GFP-NMIIB and FLAG-Aurora-B. Twenty-four hours post transfection cells were treated with DMSO or AZD for an hour, lysed, and subjected to immunoprecipitation using anti-GFP antibody. Immunoprecipitants were immunoblotted with antibodies to Phospho-Threonine and GFP. *E*, densitometry analysis of cells treated with dimethyl sulfoxide or AZD presented in *D*. Phospho-Threonine signal was normalized to the total amount of protein that was immunoprecipitated (anti-GFP signal). Error bars show the SEM from three independent experiments. *F*, HEK293T cells were cotransfected with GFP-NMIIB or GFP-NMIIB^T1847A^ and FLAG-Aurora-B. Twenty-four hours post transfection cells were lysed and subjected to immunoprecipitation using anti-GFP antibody. Immunoprecipitants were immunoblotted with antibodies to Phospho-Threonine and GFP. *G*, densitometry analysis of GFP-NMIIB and GFP-NMIIB^T1847A^ presented in *B*. Phospho-Threonine signal was normalized to the total amount of protein that was immunoprecipitated (anti-GFP signal). Error bars show the SEM from three independent experiments.

The results presented above raised the possibility that Aurora-B affects NMIIB function directly. Because Aurora-B is a serine/threonine kinase, we tested the possibility that it phosphorylates NMIIB directly. To test this possibility, we incubated Rod-B (Fig. 3*A*) with His-Aurora and subjected it to phosphorylation assay using γ-32P-ATP. Indeed, as shown in Figure S3*A*, Rod-B was phosphorylated by His-Aurora-B. Next, we attempted to map the Aurora-B phosphorylation site(s) on NMIIB. To this end, we phosphorylated Rod-B by His-Aurora-B and analyzed it with mass spectrometry. Using different proteases, such as trypsin, chymotrypsin, endoproteinase Arg-C, and endoproteinase Glu-C, to cleave the phosphorylated Rod-B produced inconsistent results. Furthermore, many peptides that were predicted to appear as the result of the protease cleavage were not detected in the mass spectrometry (data not shown). This is probably because Rod-B contains many lysine, arginine, and aspartic acid residues (62) that are the main cleavage sites of the proteases used in our study (63, 64). Therefore, the protease cleavage resulted in peptides that were too small to detect by mass spectrometry. To map the Aurora-B phosphorylation sites on NMIIB, we decided to use a different approach: we calculated the NMIIB residues score for phosphorylation by Aurora-B using the GPS software (65). The calculated scores were compared with the score obtained for threonine 117 (T^117^) in survivin, a residue that has been shown to be phosphorylated by Aurora-B and also has biological significance (66). The GPS analysis identified eight Aurora-B-specific phosphorylation sites on NMIIB, five of which reside within the tail domain of NMIIB (Fig. S3*B*). Of these five sites, three received significantly higher scores than survivin^T117^, serine 1667 (S^1667^), threonine 1762 (T^1762^), and T^1847^ (Fig. S3, *B* and *C*). We focused on T^1847^, because this residue received the highest score (Fig. S3*B*), and it is adjacent to the survivin-binding site (Fig. 3*A*). To test whether T^1847^ is indeed the main phosphorylation site, we used Rod-B as a substrate for Aurora-B because it contains the three Aurora-B phosphorylation sites with the highest scores: S^1667^, T^1762^, and T^1847^. We created Rod-B in which T^1847^ was substituted for alanine (Rod-B^T1847A^) and subjected it to phosphorylation assay with recombinant His-Aurora-B and γ-32P-ATP. As shown in Figure S3*A*, this substitution reduces the phosphorylation of Rod-B by ∼80%, indicating that T^1847^ is the main Aurora-B phosphorylation site on Rod-B. Because the phosphorylation levels of Rod-B by Aurora-B were low (Fig. S3*A*), we created several NMIIB Rod fragments and tested them for their suitability to serve as efficient substrate for Aurora-B. We found such a fragment that is composed of the last 288 amino acid residues of NMIIB (Rod-B^288^, Fig. S3*D*). As shown in Figure S3*E*, substituting T^1847^ for alanine reduced the phosphorylation levels by ∼80%, further supporting the inference that T^1847^ is the main residue phosphorylated by Aurora-B. Note that the mass spectrometry measurements did not detect any peptide containing T^1847^ (data not shown), probably because T^1847^ is surrounded by lysine and arginine amino acid residues (Fig. 3*A*). Next, we performed *in vitro* phosphorylation assay of Rod-B and Aurora-B as in Figure S3*A*, but this time we used anti-phosphothreonine antibodies to detect the phosphorylation on Rod-B. Substitution of T^1847^ to alanine reduces the phosphorylation of Rod-B by ∼80% (Fig. 3, *B* and *C*). These results are similar to the results obtained in the radioactive assay (Fig. S3*A*), indicating the validity of these results. To test whether Aurora-B phosphorylates NMIIB also *in vivo*, we analyzed the phosphorylation levels of GFP-NMIIB in the presence of AZD. As shown in Figure 3, *D* and *E*, AZD treatment reduced GFP-NMIIB phosphorylation by ∼80%. Next, we tested whether substitution of T^1847^ to alanine affects the phosphorylation levels of NMIIB *in vivo*. As shown in Figure 3, *F* and *G*, this substitution reduces the phosphorylation of NMIIB by ∼90%.

Together, these results indicate that NMIIB is a novel substrate of Aurora-B *in vitro* and *in vivo* and that the primary phosphorylation site is T^1847^.

### Phosphorylation of NMIIB by Aurora-B is important for NMIIB function in mitosis

**Figure 4:**
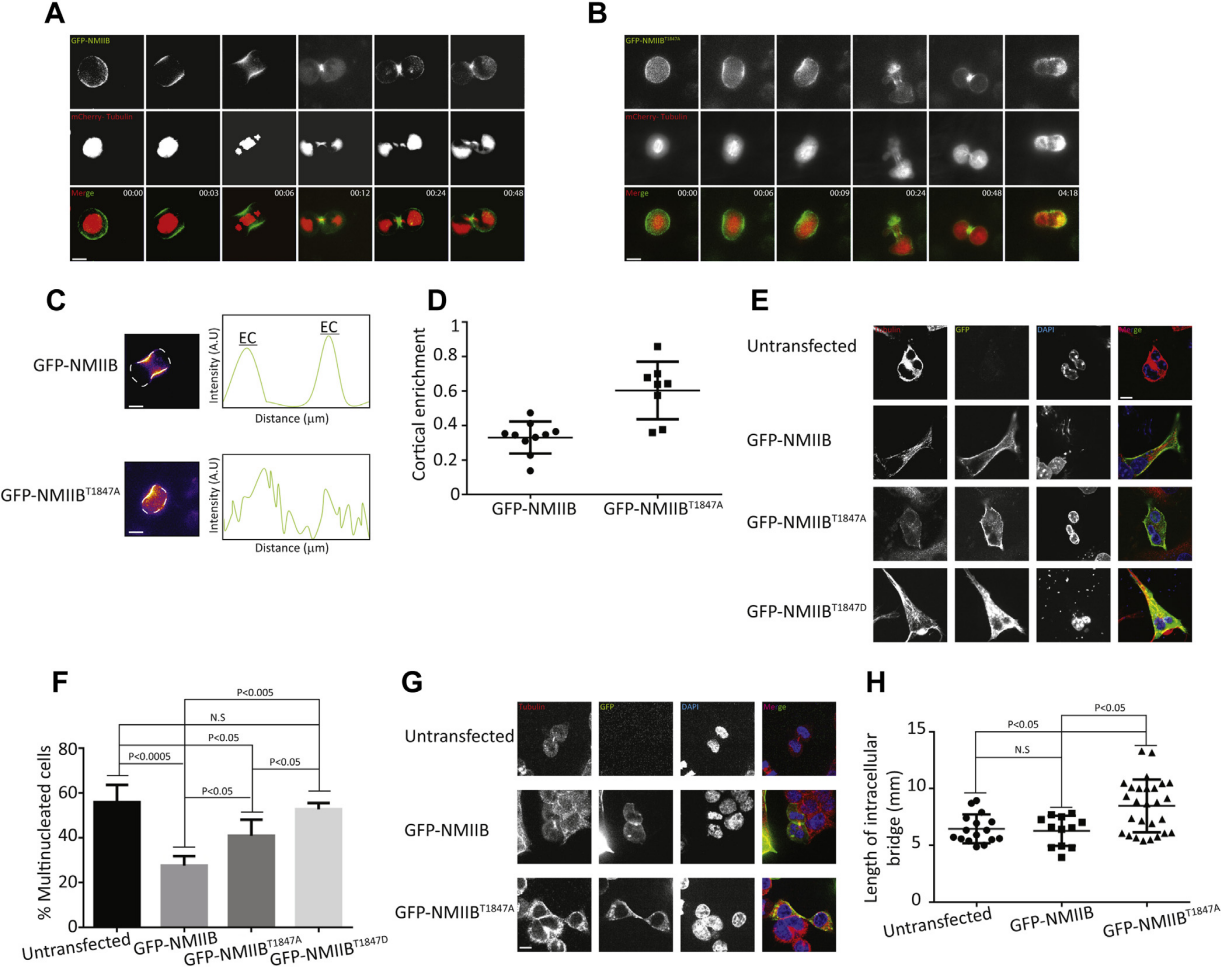
**Phosphorylation of NMIIB by Aurora-B is essential for proper mitosis.***A* and *B*, single Z-plane micrographs taken from a time-lapse movie of HeLa cells transiently expressing GFP-NMIIB (*A*) or GFP-NMIIB^T1847A^ (*B*) and mCherry-tubulin (Movies S5 and S6, respectively). Images were taken every 3 min. The scale bar represents 10 μm. *C*, *left*, fire representation of GFP-NMIIB protein localization along the cell cortex of representative mitotic HeLa cells. *Dashed line* represents the cell cortex. *Right*, line scan of GFP-NMIIB proteins along the cortex of representative mitotic HeLa cells. The scale bar represents 10 μm. *D*, quantification of GFP-NMIIB proteins cortical enrichment. Cortical enrichment was calculated by dividing the length of the cortex that is occupied by NMIIB proteins by the total length of the cell cortex. Each dot represents a cell. n = 5 and 6 for GFP-NMIIB and GFP-NMIIB^T1847A^, respectively. Statistical analysis was carried out using Student's *t* test. *E*, Cos-7 cells depleted of NMIIB were transfected with GFP-NMIIB constructs. Forty-eight hours after transfection, cells were fixed and immunostained for GFP (*green*), tubulin (*red*), and DAPI (*blue*). Shown are single Z-plane micrographs. The scale bar represents 10 μm. *F*, quantification of multinucleation in Cos-7 cells depleted of NMIIB and rescued with the indicated GFP-NMIIB constructs. Data represent mean ± SEM of three independent experiments (>30 cells/experiment and a total of at least 100 cells for each NMIIB construct were analyzed). Statistical analysis was carried out with ANOVA, followed by Tukey's test. *G*, 293T cells were transfected with the indicated GFP-NMIIB constructs. Forty-eight hours after transfection, cells were fixed and immunostained for GFP (*green*), tubulin (*red*), and DAPI (*blue*). Shown are single Z-plane micrographs. The scale bar represents 10 μm. *H*, quantification of intracellular bridge length in 293T cells transfected with the indicated NMIIB constructs. Each dot represents a cell; n = 16, 12, and 27 cells for untransfected, GFP-NMIIB, and GFP-NMIIB^T^^1847A^, respectively. Data represent mean ± SEM of three independent experiments. Statistical analysis was carried out with ANOVA, followed by Tukey's test.

To explore the importance of NMIIB phosphorylation by Aurora-B *in vivo*, we tracked the localization of GFP-NMIIB and GFP-NMIIB^T1847A^ during mitosis in live HeLa cells. The cells were also transfected with mCherry-tubulin to track the mitotic stage of the cell. GFP-NMIIB was able to concentrate at the equatorial cortex and to form the contractile ring (Fig. 4*A* and Movie S5). By contrast, GFP-NMIIB^T1847A^ localized randomly at the cell cortex and did not concentrate specifically at the equatorial cortex (Fig. 4*B* and Movie S6). Furthermore, the daughter cells of cells expressing GFP-NMIIB^T1847A^ did not separate after cytokinesis and underwent furrow regression (Fig. 4*B* and Movie S6). To further analyze the cortical localization of GFP-NMIIB^T1847A^, we measured the fluorescent intensity of GFP-NMIIB and GFP-NMIIB^T1847A^ in the cell cortex (Fig. 4*C*). GFP-NMIIB presented two fluorescent intensity peaks, representing the concentration of GFP-NMIIB at the cell cleavage furrow, whereas the GFP-NMIIB^T1847A^ fluorescent profile showed random peaks, further indicating the random localization of this protein. Note that the localization pattern of GFP-NMIIB^T1847A^ is similar to that of NMIIB in the presence of AZD (Fig. 2*H*). Next, to examine the importance of T^1847^ phosphorylation for the cortical enrichment of NMIIB, we measured the cortical index of GFP-NMIIB, as described previously (24). The cortical index represents the length of the cortical area occupied by GFP-NMIIB, divided by the length of the perimeter of the entire cell. We found that the NMIIB^T1847A^ protein presented ∼2-fold cortical enrichment compared with NMIIB, indicating the importance of Aurora-B phosphorylation of NMIIB for its dynamic cortical localization during early telophase (Fig. 4, *C* and *D*).

Next, to determine whether NMIIB phosphorylation by Aurora-B is also important in late telophase/cytokinesis, we tested the ability of GFP-NMIIB phosphomutants to rescue multinucleation in Cos-7 cells caused by NMIIB depletion (19). The Cos-7 cell line does not express endogenous NMIIA (57), and depletion of NMIIB causes mitotic defects (19). We transfected Cos-7 cells depleted of NMIIB with GFP-NMIIB^T1847A^ or GFP-NMIIB^T1847D^ and tested the ability of these proteins to rescue multinucleation. As shown in Figure 4, *E* and *F*, GFP-NMIIB transfected cells reduced the number of multinucleated cells from 70% to 25%. GFP-NMIIB^T1847A^ and GFP-NMIIB^T1847D^ expression had significantly less of an effect on the percentage of multinucleated cells: 45% and 55%, respectively. These results indicate that regulation of NMIIB by Aurora-B phosphorylation is indispensable for proper cytokinesis.

Another phenomenon that we detected in Cos-7 cells depleted of NMIIB and expressing NMIIB^T1847D^ was that NMIIB^T1847D^ had a diffuse pattern (Fig. 4*E*), which may indicate its inability to assemble into filaments. We further found that, in Cos-7 cells depleted of NMIIB and expressing NMIIB^T1847A^, GFP-NMIIB^T1847A^ localized to the midbody and intercellular bridge, a region that is not usually occupied by this protein (Fig. S4, *A* and *B*). We observed the same phenomenon also in HEK293T cells expressing GFP-NMIIB^T1847A^ (Fig. S4, *C* and *D*). Because the exclusion of actomyosin components from the midbody is essential for proper cytokinesis (67), and because Aurora-B activity is at its peak at the equatorial cortex during later stages of mitosis (61), we hypothesized that NMIIB phosphorylation by Aurora-B is important for proper midbody formation. To test this hypothesis more rigorously, we transfected HEK 293T cells with GFP-NMIIB and GFP-NMIIB^T1847A^ and measured the length of the intercellular bridge in these cells. We conducted these experiments on HEK 293T because these cells have a propensity for transfection, and therefore we can obtain a large population of cells expressing GFP-NMIIB or GFP-NMIIB^T1847A^. As shown in Figure 4, *G* and *H*, the length of the intercellular bridge of cells expressing GFP-NMIIB was comparable with the intercellular bridge length of untransfected cells (7 μm in both cell populations). By contrast, the intercellular bridge length of cells expressing GFP-NMIIB^T1847A^ was significantly higher (10 μm) (Fig. 4, *G* and *H*). Moreover, some of these cells showed an extremely long intercellular bridge, which we termed "extra-elongated intercellular bridge" (Fig. S4*D*). Note that cells with the extra-elongated intercellular bridge were not included in the analysis presented in Figure 4*H*. Overall, these results indicate that GFP-NMIIB^T1847A^ is mislocalized at the midbody and intercellular bridge and causes cytokinesis defects, supporting the findings that Aurora-B phosphorylation of NMIIB plays a crucial role in cytokinesis.

### NMIIB phosphorylation by Aurora-B modulates NMIIB filament assembly properties

**Figure 5:**
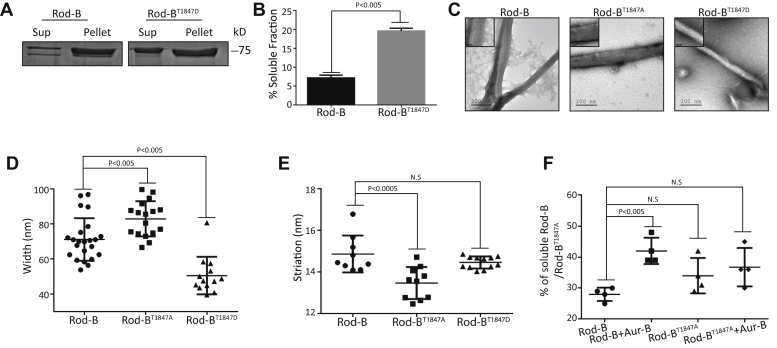
**T**^**1847**^**phosphorylation affects Rod-B filament assembly and paracrystal structure**. *A*, Rod-B and Rod-B^T1847D^ were subjected to filament assembly assay and analyzed on Coomassie-stained SDS-PAGE gels. *B*, the extent of filament assembly, shown in *A*, was quantified by calculating the amount of Rod-B in the supernatant (sup) and pellet. Data represent mean ± SEM of three independent experiments. A two-tailed Student's *t* test was used for statistical analysis. *C*, electron micrographs of paracrystals formed by Rod-B proteins that were expressed and purified from *E. coli*, dialyzed against low NaCl buffer, and stained with uranyl acetate before viewing by electron microscope. Insets are enlarged electron micrographs showing filament striations. The scale bar represents 50 nm. *D*, the width of Rod-B paracrystals was measured using Image J. Each dot represents a Rod-B filament; n = 23, 17, and 13 for Rod-B, Rod-B^T1847A^, and Rod-B^T1847D^, respectively. Data represent mean ± SEM of two independent experiments. Statistical analysis was carried out with ANOVA, followed by Tukey's test. *E*, the striation of Rod-B paracrystals was measured using Image J. Each dot represents a Rod-B filament; n = 9, 10, and 12 for Rod-B, Rod-B^T1847A^, and Rod-B^T1847D^, respectively. Data represent mean ± SEM of two independent experiments. Statistical analysis was carried out with ANOVA, followed by Tukey's test. *F*, quantification of the percentage of Rod-B in supernatant after Aurora-B phosphorylation. Rod-B or Rod-B^T1847A^ were subjected to phosphorylation assay with Aurora-B. The protein mix was subjected to high-speed centrifugation. The percent of Rod-B or Rod-B^T1847A^ in the soluble fraction was calculated as in B. Data represent mean ± SEM of four independent experiments. Statistical analysis was carried out with ANOVA, followed by Tukey's test.

Previous studies identified regions along the NMII tail that are critical for filament formation. A highly conserved region near the C-terminal end of NMII was termed “the assembly competence domain” or ACD (Fig. 3*A*) (68, 69, 70). Previous studies in our laboratory have identified a region N-terminal to ACD, also important for filament assembly, which we termed “complementary ACD” (cACD, Fig. 3*A*; (70)). Because T^1847^ resides within the cACD (Fig. 3*A*), we hypothesized that Aurora-B phosphorylation of NMIIB may affect the properties of NMIIB filament assembly. To test this hypothesis, we created a phosphomimetic Rod-B where T^1847^ was substituted for aspartic acid (Rod-B^T1847D^) and subjected it to filament assembly assay. Rod-B^T1847D^ was significantly less competent to form filaments than was Rod-B (Fig. 5*A*). Only 6% of Rod-B did not assemble into filaments, whereas 20% of Rod-B^T1847D^ was not capable of doing so (Fig. 5*B*). These results indicate that Aurora-B phosphorylation of NMIIB hinders filament assembly.

NMII forms ordered filamentous structures called paracrystals, which have a distinct pattern of striations similar to bipolar filaments seen *in vivo* (13, 62, 71). To explore further the role of Aurora-B phosphorylation in NMIIB filament assembly, we inspected the morphology of paracrystals formed by Rod-B^T1847A^ and Rod-B^T1847D^. Rod-B formed thick filaments with distinct striation (Fig. 5*C*), as reported previously (13, 14, 62, 71). Rod-B^T1847A^ formed wider filaments than those formed by Rod-B (82.9 versus 71.13 nm) (Fig. 5, *C* and *D*). In addition, Rod-B^T1847A^ exhibited an aberrant striation pattern. Rod-B formed ordered filaments with the expected striation pattern of 14.84 nm, whereas Rod-B^T1847A^ had a smaller striation pattern of 13.48 nm (Fig. 5, *C* and *E*), suggesting impaired packing of Rod-B^T1847A^ filaments. Although the striation pattern of Rod-B^T1847D^ filaments was similar to that of Rod-B (14.47 versus 14.84 nm) (Fig. 5, *C* and *E*), Rod-B^T1847D^ filaments were significantly thinner than the filaments formed by Rod-B (50.53 versus 71.13 nm) (Fig. 5, *C* and *D*). Together, these results indicate that Aurora-B phosphorylation abrogates the ability of NMIIB to form filaments and affects the structure of the paracrystals. Thus, the NMIIB filament assembly is regulated by Aurora-B phosphorylation of T^1847^.

Next, we tested whether Aurora-B phosphorylation of Rod-B in filaments affects the integrity of the filaments. We incubated Rod-B and Rod-B^T1847A^ with His-Aurora B and subjected them to phosphorylation assay. Note that the phosphorylation conditions were similar to those of Rod-B paracrystals formation. Under these conditions, ∼28% and ∼34% of Rod-B and Rod-B^T1847A^ were in the nonfilamentous fraction, respectively (Fig. 5*F*). To ensure that the Rod-B proteins were phosphorylated, we performed in parallel a phosphorylation assay using His-Aurora-B and γ-32P-ATP. As shown in Figure 5*F*, phosphorylation of Rod-B filaments by Aurora-B increased the nonfilamentous fraction of Rod-B from 28% to 42% (Fig. 5*F*). By contrast, Aurora-B phosphorylation of Rod-B^T1847A^ did not have any significant effect on its filament integrity (34% without His-Aurora-B versus 36.75% with His-Aurora-B) (Fig. 5*F*). These results indicate that Aurora-B phosphorylation of Rod-B filaments leads to filament disassembly and that T^1847^ is the main Aurora-B phosphorylation site.

### Aurora-B phosphorylation of NMIIB interferes with NMIIB–survivin interactions

**Figure 6:**
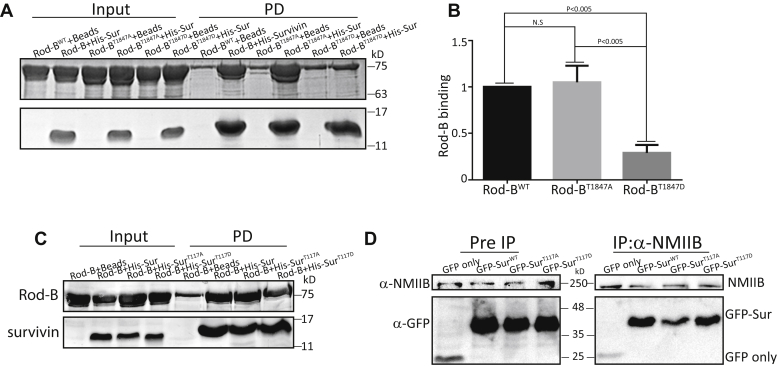
**Aurora-B phosphorylation of NMIIB regulates the interaction between NMIIB and survivin.***A*, Coomassie-stained SDS-PAGE of His-survivin and Rod-B, Rod-B^T1847A^, and Rod-B^T1847D^ subjected to a pull-down assay. Ni^2+^-NTA beads served as a negative control. *B*, quantification of the pull-down assay shown in A. The extent of Rod-B^T1847A^ and Rod-B^T1847D^ binding to survivin relative to Rod-B is plotted. Data represent mean ± SEM of three independent experiments. ANOVA followed by Tukey's test was used for statistical analysis. *C*, Coomassie-stained SDS-PAGE of Rod-B and His-survivin, His-survivin^T117A^, and His-survivin^T117D^ subjected to a pull-down assay. Ni^2+^-NTA beads served as a negative control. *D*, GFP-survivin, GFP-survivin^T117A^, and GFP-survivin^T117D^ were expressed in 293T cells and coimmunoprecipitated with endogenous NMIIB, using anti-NMIIB antibody. The immunoprecipitated proteins were analyzed by immunoblotting with antibodies against NMIIB and GFP. GFP alone was used as a negative control.

Survivin and Aurora-B are part of the CPC (33). Aurora-B phosphorylates survivin, regulating its function during mitosis (66). Recently, we showed that survivin regulates the spatiotemporal formation of the acto-NMII contractile ring during cytokinesis (19). Thus, it is plausible that Aurora-B phosphorylation of NMIIB affects NMIIB interaction with survivin. To test this possibility, we subjected Rod-B and Rod-B phosphomutants to a direct pull-down assay using recombinant survivin. Rod-B and Rod-B^T1847A^ were able to bind survivin, whereas Rod-B^T1847D^ showed 80% reduction in its ability to do so (Fig. 6, *A* and *B*). Thus, phosphorylation of NMIIB by Aurora-B regulates its interaction with survivin.

It was previously reported that survivin is phosphorylated by Aurora-B on T^117^ (66). Further studies showed that this phosphorylation is essential for the mitotic functions of survivin (66, 72, 73). Moreover, it was shown that the survivin phosphomimetic mutant (survivin^T117E^) was not able to compensate for the depletion of endogenous survivin, resulting in cytokinesis defects (66). Nevertheless, the reason for the inability of survivin^T117E^ to rescue cells depleted of survivin is not fully understood. Therefore, we investigated the possibility that Aurora-B regulates the interaction of survivin with NMIIB by phosphorylating survivin. To this end, we generated survivin proteins mutated at the Aurora-B phosphorylation site, survivin^T117A^ and survivin^T117D^, respectively, and subjected them to a direct pull-down assay with Rod-B. Survivin^T117A^ and survivin^T117D^ were capable of binding Rod-B to an extent similar to survivin (Fig. 6*C*). To verify these results *in vivo*, we transfected HEK 293T cells with GFP-survivin phosphor-mutants and subjected them to a coimmunoprecipitation assay with endogenous NMIIB, using anti-NMIIB antibodies. Consistent with the results obtained *in vitro*, the phosphor-mutant survivin proteins formed a complex with NMIIB *in vivo* (Fig. 6*D*). Together, these results indicate that survivin phosphorylation by Aurora-B does not affect its ability to form a complex with NMIIB. These results suggest that the mitotic significance of survivin^T117^ is probably not carried out by altering the function of NMIIB in mitosis.

## Discussion

It has been known for many years that many cytoskeletal proteins, such as NMII, are essential for cell division. NMII is a key component of the contractile ring, a structure that is essential to achieve cytokinesis (74). Despite growing knowledge about the role of NMII in cytokinesis, many questions remained open. For example, how is the acto-NMII recruited to the equatorial cortex and excluded from other regions of the cell cortex during anaphase and telophase? How is the assembly of the contractile ring regulated? How is the acto-NMII discarded from the midbody during cytokinesis? What is the mechanism of the contractile ring disassembly before abscission?

Aurora-B kinase is an important factor in mitosis. Aurora-B phosphorylates many substrates through mitosis (40), and its activity is essential for executing the mitosis; therefore, it must be strictly regulated. On the one hand, inhibition of Aurora-B, either genetically (dominant negative mutation) or chemically (using a small molecule), causes mitotic defects (75). On the other hand, overexpression of Aurora-B also causes mitotic defects (such as aneuploidy) (41), pointing to the importance of the timing of substrate phosphorylation by Aurora-B during mitosis. Thus, Aurora-B substrate phosphorylation during mitosis must be highly regulated in time and space.

The data we present here may assist in answering some of the questions concerning the regulation of NMII during mitosis. We report on a novel Aurora-B substrate, NMIIB. Aurora-B and NMIIB form a complex *in vivo*, which is independent on Aurora-B kinase activity (Fig. 1, *B*–*E*). Furthermore, we show that the Aurora-B–NMIIB complex includes survivin homodimer and not survivin monomer (Fig. 1, *F*–*I*). Nevertheless, survivin dimerization mutants form a complex with other CPC members, such as Aurora-B and Borealin, indicating that in the CPC survivin resides as a monomer. These results are in agreement with structural studies (34). Our findings suggest that there are at least two CPC pools, one that contains survivin monomer and another that contains survivin homodimer that also binds NMIIB (Fig. 1*E*). Recently, two mechanisms coordinating CPC recruitment to the cell division plane were identified, suggesting that the CPC may form different complexes with different stoichiometry (76).

We show that, upon Aurora-B inhibition or expression of NMIIB^T1847A^, NMIIB and NMIIB^T1847A^ are highly enriched in the cell cortex (Fig. 2, *E*–*H*), an indication of NMIIB overassembly that can be attributed to excessive activity of NMIIB. Thus, Aurora-B is an inhibitor of NMIIB. These results agree with those of studies showing that during cytokinesis the region of the NMII activator RhoA is expanded upon Aurora-B inhibition (39), indicating that Aurora-B inhibits RhoA and NMII activity. Studies on *C. elegans,* however, indicate that Aurora-B activates RhoA, which may lead to NMII activation (37). These seemingly contradicting results may point to the complexity of the mechanisms by which Aurora-B regulates different aspects of mitosis.

Our data imply that Aurora-B phosphorylates NMIIB *in vivo* and *in vitro* on T^1847^, a residue that is in close proximity to the binding site of survivin (Fig. 3*A* and (19)). The mass spectrometry studies did not identify T^1847^ as a phosphorylated residue, nor did other studies that investigated mitotic phosphor-proteomics identify T^1847^ as a mitotic phosphor residue (77, 78, 79). This discrepancy may be explained by the primary structure of NMII and the method by which mass spectrometry preparations are made. The proteases that are used cleave at arginine, aspartate, and lysine, amino acid residues that are common in the NMII Rod. Thus, the protease cleavage produces extremely small peptides that cannot be detected by mass spectrometry. Indeed, T^1847^ is surrounded by arginine, aspartate, and lysine; therefore, the peptides obtained by protease cleavage are too small to be detected.

Using GPS software (65), combined with *in vivo* and *in vitro* Aurora-B phosphorylation assays, we identified T^1847^ as the main site of Aurora-B phosphorylation (Fig. 3). We showed that inhibition of Aurora-B by AZD or expression of GFP-NMIIB^T1847A^ significantly reduced the phosphorylation of NMIIB *in vivo* (Fig. 3, *D*–*G*), Rod-B served as a substrate for Aurora-B in the phosphorylation assays, but we found that a shorter fragment of Rod-B, Rod-B^288^, was an efficient substrate and we were able to determine with a high degree of confidence that T^1847^ is the main phosphorylation site of Aurora-B. These findings raise the question why Rod-B^288^ is a better substrate than Rod-B. Although both proteins contain the important domains for proper filament assembly (*i.e.*, ACD and cACD, Fig. 3*A*), we found that Rod-B^288^ filaments are less packed than those of Rod-B (70). We speculate that Rod-B^288^ is more accessible to Aurora-B than is Rod-B; therefore, Rod-B^288^ is phosphorylated by Aurora-B to a greater extent than Rod-B is. We attempted to perform a phosphorylation assay using a high-salt-induced Rod-B disassembly (*i.e.*, Rod-B monomers), but Aurora-B was inactive under these conditions (data not shown). We propose that the Rod-B^288^ filament state mimics to some extent the state of NMIIB filaments *in vivo*. It was shown that NMII forms small filaments, referred to as minifilaments, in the contractile ring of mammalian cells (80, 81). Thus, NMIIB minifilaments are accessible for phosphorylation by Aurora-B *in vivo*. Phosphorylation of Rod-B by Aurora-B reduced the ability of Rod-B to form filaments and altered paracrystal properties (Fig. 5). More important, we showed that Aurora-B phosphorylation of Rod-B filaments leads to filament disassembly (Fig. 5*F*). This raises the possibility that Aurora-B plays a role in contractile ring disassembly before abscission.

The importance of Aurora-B phosphorylation of NMIIB in the regulation of NMIIB in mitosis is revealed by the behavior of NMIIB phosphomutants during mitosis. GFP-NMIIB^T1847A^ and GFP-NMIIB^T1847D^ were unable to fully rescue the phenotype of Cos-7 cells depleted of NMIIB (Fig. 4, *E* and *F*). The fact that neither mutant was able to fully rescue cytokinesis defects further supports the notion that the phosphorylation status of Aurora-B substrates should be highly regulated. Of interest, NMIIB^T1847D^ in Cos-7 cells had a diffuse pattern (Fig. 4*E*), which may indicate its inability to assemble into filaments. Although Rod-B^T1847D^ was capable of forming filament with striation similar to Rod-B, it was less competent to assemble into filaments (Fig. 5, *A* and *B*) and these filaments were thinner than the filaments formed by Rod-B (Fig. 5*D*). Thus, the effect of phosphomimetic mutation on the ability of NMIIB (NMIIB^T18^^4^^7D^) to assemble into filaments is significantly more robust than that of phosphomimetic Rod-B (Rod-B^T1847D^). This difference can be explained by the findings that filaments formed *in vitro* by myosin II rods contain several hundreds of rod molecules (82), whereas filaments that are formed *in vivo* by myosin II contain less than 30 molecules (83).

The importance of NMIIB regulation by Aurora-B was further illustrated by HEK293 cells expressing GFP-NMIIB^T1847A^. These cells present a long intercellular bridge, indicating that they have abscission defects (Fig. 4, *G* and *H*). Note that these defects also include the presence of GFP-NMIIB^T1847A^ in the intercellular bridge, a region that is not usually occupied by NMII, further supporting the idea that some cytoskeletal proteins must be excluded from the intercellular bridge for abscission to take place (67). Note further that similar abscission defects were shown for other substrates of Aurora-B, such as vimentin and keratin 5 (59, 84). Thus, Aurora-B regulates abscission through phosphorylation of different substrates.

The heavy chains of NMIIB are phosphorylated by several protein kinases, such as PKCγ and aPKCζ (10, 24, 85). For example, aPKCζ phosphorylates NMIIB Serine^1935^ in response to EGF stimulation, regulating NMIIB filament assembly and cellular organization (85). Furthermore, NMIIB Serine^1935^ was shown to regulate the role of NMIIB in migratory front–back polarity (26). We propose that different kinases phosphorylate NMIIB in response to different signals, thus regulating the distinct functions of NMIIB in cell migration and cytokinesis.

We have previously shown that survivin forms a complex with NMIIB *in vivo* through direct interaction (19). Moreover, the survivin–NMIIB complex is regulated by phosphorylation of survivin by CDK1 on threonine 34. Our results indicate that phosphorylation of NMIIB by Aurora-B regulates its interaction with survivin. Thus, Aurora-B phosphorylation of NMIIB regulates NMIIB-survivin complex formation as well as NMIIB filament integrity. Survivin phosphorylation by Aurora-B, however, does not affect NMIIB–survivin complex formation, suggesting that Aurora-B regulates survivin in mitosis in a process different from the process involving the NMIIB–survivin complex. Understanding what process is regulated by Aurora-B-dependent survivin phosphorylation is beyond the scope of this study and requires further investigation.

**Figure 7:**
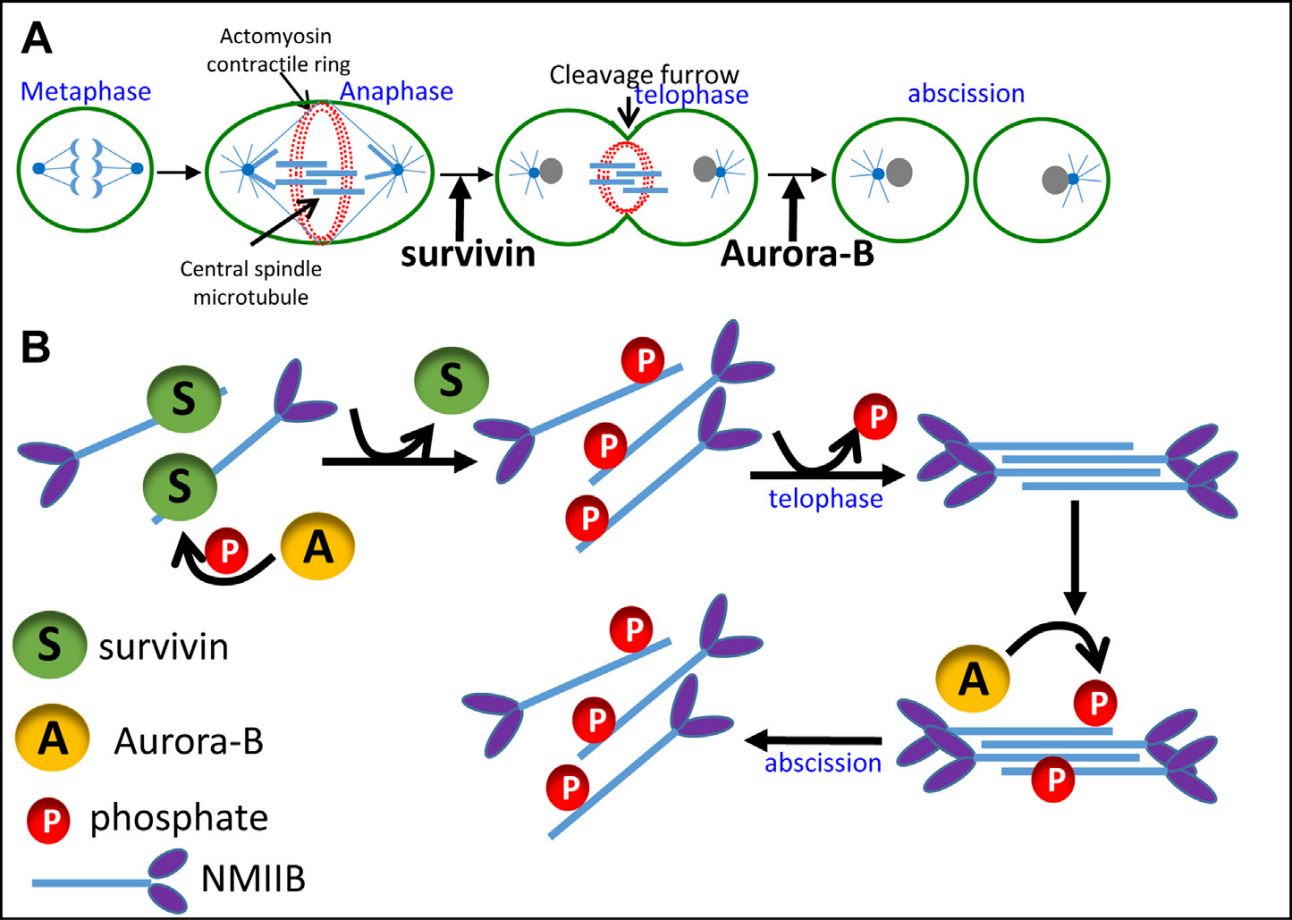
**A model depicting the role of Aurora-B in the regulation of NMII in mitosis.***A*, the stages of mitosis where NMIIB, Aurora-B, and survivin function. *B*, the mechanism by which Aurora-B and survivin regulate NMIIB during mitosis. Upon onset of anaphase, NMIIB forms a complex with survivin regulating the rate of NMIIB filament formation and their cellular localization. As mitosis progresses, NMIIB is phosphorylated by Aurora-B at T^1847^, releasing NMIIB from survivin. This is followed by NMIIB being dephosphorylated by an as yet unknown phosphatase, allowing NMIIB to assemble into filaments and form the contractile ring. At the end of cytokinesis, Aurora-B phosphorylates NMIIB, leading to contractile ring disassembly.

Based on the data reported here, we propose a model for the regulation of NMIIB by Aurora-B (Fig. 7). We propose that, during anaphase, survivin interacts with NMIIB at the equatorial cortex (19), regulating the rate and localization of NMIIB filament assembly during contractile ring formation (19) (Fig. 7*A*). We further propose that Aurora-B has several novel functions during the late stages of mitosis (Fig. 7, *A* and *B*). First, Aurora-B regulates the contractile ring formation through the regulation of NMIIB–survivin complex formation. We propose that Aurora-B phosphorylates NMIIB, releasing it from survivin inhibition, and upon phosphate removal from NMIIB, it assembles into filaments at the contractile ring (Fig. 7*B*). Second, Aurora-B regulates contractile ring assembly during telophase, by regulating the proper localization of NMIIB. This is supported by the findings that Aurora-B inhibition causes NMIIB overassembly and abnormal cortical localization. Phosphorylation during mitosis is a dynamic process (86); therefore, it is plausible that Aurora-B phosphorylation plays a role in "fine-tuning" the contractile ring assembly. Finally, in abscission Aurora-B rephosphorylates NMIIB, leading to NMIIB filament disassembly (Fig. 7, *A* and *B*). Thus, Aurora-B phosphorylates NMIIB in the course of two different events: the first phosphorylation removes survivin from NMIIB, followed by dephosphorylation, and the second phosphorylation disassembles the NMIIB filaments (Fig. 7). This hypothesis is supported by the fact that several phosphatases participate in cytokinesis in many organisms (87), and there is an extensive interplay between kinases and phosphatases during mitosis (86). Therefore, it is possible that NMIIB is phosphorylated by Aurora-B more than once during the cell cycle.

It has been recently shown that NMII contractility and Aurora-B kinase functions are important for a stable midzone (88). Midzone microtubules were less stable in cells treated with NMII or Aurora-B inhibitors. It is plausible, therefore, that there are other mechanisms by which the interplay between Aurora-B and NMIIB regulates mitosis.

Many studies have shown an interplay between the cytoskeleton and Aurora-B during mitosis. For instance, the mitotic kinesin-like protein 2 (MKLP2) plays a role in transporting the CPC during mitosis (76, 89). These studies showed that the cytoskeleton is essential for Aurora-B localization. It has also been shown that Aurora-B activity causes local disassembly of actin and keratin in *Xenopus laevis* eggs (90) and that Aurora-B phosphorylates the myosin light chain (55). Thus, Aurora-B is important for proper cytoskeleton function during mitosis. Other studies showed further that direct phosphorylation of cytoskeletal proteins, such as keratin 5, by Aurora-B is essential for proper mitosis (84). Aurora-B also regulates abscission by phosphorylating MKLP2 (45). Our results extend the knowledge regarding Aurora-B and the cytoskeleton cross-talk during mitosis.

Nevertheless, many issues remain to be addressed. For example, we showed that Aurora-B prevents NMIIB from accumulating in random areas of the cortex, but it is not known who is responsible for recruiting NMIIB specifically to the equatorial cortex. Some data suggest that MKLP2 interacts with both Aurora-B and NMII (91), so that MKLP2 might be the motor that localizes NMII and Aurora-B at the cell cortex. Another question that requires further investigation concerns the identity of the phosphatase(s) that dephosphorylate NMIIB during mitosis. B56-PP2A is a possible candidate, as this phosphatase has been shown to counteract Aurora-B activity during late mitosis (45).

## Materials and methods

### Cell lines and culture conditions

HeLa, Cos-7, and HEK293T cell lines were purchased from the American Type Culture Collection and were maintained in high-glucose Dulbecco's modified Eagle's medium (Sigma-Aldrich) supplemented with 2 mM L-glutamine, 10% fetal calf serum, and antibiotics (100 U/ml penicillin, 100 mg/ml streptomycin, and 1:100 Biomyc3 anti-mycoplasma antibiotic solution; Biological Industries). Cells were grown at 37 °C in a humidified atmosphere of 5% CO_2_ and 95% air.

### Antibodies

Antibodies specific for the C-terminal region of human NMIIB (used at 1:1000) were generated in rabbits according to the method of (92). Recombinant GFP antibodies (used at 1:1000 dilution for Western blot and 1:200 for immunofluorescence) were prepared in rabbits as described (24). Rabbit polyclonal anti-Aurora-B antibodies (ab2254, 1:1000 for Western blot and 1:200 for immunofluorescence), mouse monoclonal anti-NMIIB (ab684, 1:200), and rat monoclonal anti-tubulin (ab6160, 1:200) were purchased from Abcam. Mouse monoclonal anti-FLAG (F3165, 1:1000) was purchased from Sigma-Aldrich. Mouse monoclonal anti-Phospho-Threonine (catalog no. 9386, clone 42H4, 1:500) was purchased from Cell Signaling Technology. Horseradish peroxidase–conjugated secondary antibodies, donkey anti-rat-IgG conjugated to Alexa Fluor 555, goat anti-mouse-IgG conjugated to Alexa Fluor 488, goat anti-rabbit-IgG conjugated to Cy5, and goat anti-mouse conjugated to Cy5 were from Jackson ImmunoResearch Laboratories.

### Construction of NMIIB mutants

All primers used for plasmid constructions are presented in Table 1. Restriction enzymes were from New England Biolabs or Fermentas. To create Rod-B^T1847A^, pET21C-Rod-B (62) was subjected to three-step PCRs. The first and second PCRs were carried out with primers #1 and #4 and primers #2 and #3, respectively (Table 1). The PCR products were subjected to a third PCR reaction using primers #1 and #2. The resulting PCR product was digested with BamHI and EcoRI and ligated into pET21C digested with the same enzymes. For Rod-B^T^^1847D^ construction, pET21C-Rod-B was subjected to a three-step PCR, the first and second PCRs with primers #1 and #6, and primers #2 and #5, respectively (Table 1). The PCR products were subjected to a third PCR reaction with primers #1 and #2. The resulting PCR product was digested with BamHI and EcoRI and ligated into pET21C digested with the same enzymes. To create GFP-NMIIB mutant constructs, pET21C-Rod-B^T1847A^ and pET21C-Rod-B^T1847D^ were digested with SmaI and ligated into eGFP-NMIIB-C3 (kindly provided by Dr Robert S. Adelstein, Laboratory of Molecular Cardiology, NIH) digested with SmaI. Cloning of Rod-B^288^ was described previously (70). To create Rod-B^288 T1847A^, pET21C-Rod-B^288^ was subjected to three-step PCRs. The first and second PCRs were carried out with primers #1 and #4 and primers #2 and #3, respectively (Table 1). The PCR products were subjected to a third PCR reaction using primers #1 and #2. The resulting PCR product was digested with BamHI and EcoRI and ligated into pET21C digested with the same enzymes.

**Table 1:** Primers used in this study

Primer	Sequence
1	5′-GGC GGC TGC TCG TTC CTT GGC CAG CTG CCC AAT CTT GGC CTC-3′
2	5′-GCT AGT TAT TGC TCA GCG G-3′
3	5′-CAA ATT AGT CCG TCG CGC TGA GAA GAA GCT GAA AG-3′
4	5′-CTT TCA GCT TCT TCT CAG CGC GAC GGA CTA ATT TG-3′
5	5′-CAA ATT AGT CCG TCG CGA TGA GAA GAA GCT GAA AG-3′
6	5′-CTT TCA GCT TCT TCT CAT CGC GAC GGA CTA ATT TG-3′
7	5′-TCG ACC TGC AGC CAA GCT-3′
8	5′-GGA TCC GTG ATG GTG ATG-3′
9	5′-TAT GGA GAA TCT TTA CTT TCA GGG GGC CCA GAA GGA GAA CTC C-3′
10	5′-GGC TTT GTT AGC AGC CGG ATC CTC GAG TCA GGC GAC AGA TTG AAG GGC-3′
11	5′-AAT TAG ATC TAT GGC CCA GAA GGA GAA CTC CTA C-3′
12	5′-AAT TGA ATT CTC AGG CGA CAG ATT GAA GGG CAG-3′
13	5′-AAT TGA ATT CCA TGG CCC AGA AGG AGA AC-3′
14	5′-AAT TAG ATC TTC AGG CGA CAG ATT GAA G-3′
15	5′-TTC ATC GTG GCG CTC AGG GTC CTC TTC AAG TCC-3′
16	5′-GGA CTT GAA GAG GAC CCT GAG CGC CAC GAT GAA-3′
17	5′-TAG GAT CCG GTG CCC CGA CGT TGC-3′
18	5′-TAG AAT TCT CAA TCC ATG GCA GCC AGC TG-3′
19	5′-GCC AAG AAC AAA ATT GCA AAG GAA GCC AAC AAT AAG AAG AAA GAA T-3′
20	5′-ATT CTT TCT TCT TAT TGT TGG CTT CCT TTG CAA TTT TGT TCT TGG C-3′
21	5′-ATT CTT TCT TCT TAT TGT TGT CTT CCT TTG CAA TTT TGT TCT TGG C-3′
22	5′-GCC AAG AAC AAA ATT GCA AAG GAA GAC AAC AAT AAG AAG AAA GAA T-3′
23	5′-GGA CTC AGA TCT GGT GCC CCG ACG TTG CCC CCT-3′

### Construction of Aurora-B

Cloning 6XHis-Aurora-B was undertaken using the Gibson assembly method (93). PET15B vector was subjected to PCR using primers #7 and #8 (Table 1). GFP-Aurora-B (a gift from Dr Susanne M. A. Lens, Department of Medical Oncology, University Medical Center Utrecht) was subjected to PCR using primers #9 and #10 (Table 1). The PCR products were digested with DpnI and assembled with Gibson assembly master mix (New England Biolabs) according to the manufacturer's instructions. To clone Aurora-B into pEGFP-C1, or pmCherry-C1, BglII and EcoRI restriction sites were added to GFP-Aurora-B using primers #11 and #12 (Table 1). The PCR products were digested with BglII and EcoRI and ligated into pEGFP-C1 or pmCherry-C1 digested with the same enzymes. To create FLAG-Aurora-B EcoRI and BglII restriction sites were added to GFP-Aurora-B using primers #13 and #14 (Table 1). The PCR products were digested with EcoRI and BglII and ligated into pFLAG-CMV-2 digested with the same enzymes. To create GFP-Aurora-B^K106R^, GFP-Aurora-B was subjected to a three-step PCR, the first and second PCRs were carried out using primers #15 and #12, and primers #16 and #11 (Table 1), respectively. The resulting PCR products were subjected to a third PCR with primers #11 and #12 (Table 1), and the final PCR product was digested with BglII and EcoRI and ligated into pEGFP-C1 digested with the same enzymes.

### Construction of survivin mutants

pcDNA-survivin was kindly provided by Dr Sally Wheatley (University of Nottingham). The creation of His-survivin, His-survivin^L6A/W10A^, GFP-survivin, and GFP-survivin^L6A/W10A^ was described (19).

To create His-survivin^T117A^, a three-step PCR was performed, primers #19 and #18, and #20 and #17 (Table 1) were used for the first and second PCRs, respectively. The resulting PCR products were subjected to a third PCR reaction with primers #9 and #10 (Table 1). The final PCR product was digested with BamHI and EcoRI and ligated into pQE80L digested with the same enzymes. A similar three-step PCR was used to create His-survivin^T117D^. The first and second PCRs were carried out with primers #17 and #21, and primers #18 and #22 (Table 1), respectively. The resulting PCR products were subjected to a third PCR with primers #9 and #10 (Table 1), and the final PCR product was digested with BamHI and EcoRI and ligated into pQE80L digested with the same enzymes.

To clone GFP-tagged survivin proteins, BglII and EcoRI restriction sites were added to pQE80L containing the survivin constructs using primers #18 and #23 (Table 1).

Generation of the inducible Cos-7 NMIIB^KD^ cell line was described (19).

### Protein expression and purification

Rod-B proteins were purified as described (62). His-Aurora-B was grown in the *E. coli* T7+ strain to an absorbance at 600 nm (*A*600nm) of 0.5, and 0.5 mM isopropyl-β-d-thiogalactoside (IPTG) was added and the bacteria were grown at 16 °C overnight. Bacterial pellets were collected by centrifugation at 10,000*g* (Sorvall Thermo-Scientific, Rotor F12) and dissolved in Buffer A containing 50 mM Tris-HCl pH 8, 500 mM NaCl, glycerol 1%, 20 mM imidazole, 20 mM β-mercaptoethanol, 0.5 mM phenylmethylsulfonyl fluoride (PMSF), and 1% Tween-20. Bacterial suspensions were sonicated and centrifuged using F21-8×50y rotor (Thermo-Scientific) at 20,000*g* for 15 min. Supernatants were collected and loaded on a Ni^2+^-NTA bead column (GE Healthcare) prewashed with Buffer A without PMSF. His-Aurora-B was eluted with Buffer A without PMSF containing 250 mM imidazole. Fractions containing proteins were pooled and dialyzed against Buffer A without imidazole, and protein concentration was determined with a Nanodrop spectrophotometer.

### Coimmunoprecipitation assay

A total of 1–2 × 10^6^ HEK293T cells were seeded on a 60-mm dish. After attaching to the dish (10–24 h), cells were transfected with 6 μg DNA per plate, mixed with 36 μg linear PEI (L.PEI). Cells were harvested at 24 to 48 h post transfection with 300 μl extraction buffer (20 mM Tris-HCl pH 8.0, 225 mM NaCl, 0.5 mM EDTA, 1% NP-40, 5% glycerol,1 mM DTT, and protease inhibitor cocktail [Sigma-Aldrich]). Cell extracts were sonicated and centrifuged at 4 °C for at least 15 min (>16,000*g*). Anti-NMIIB antibodies were incubated with protein A/G beads (Santa Cruz Biotechnology) prewashed with 300 μl extraction buffer on a rotator at 4 °C for 1.5 to 2 h. The beads–antibodies mix was washed three times with extraction buffer. The cell extracts were added to the bead–antibody mix and were incubated for 1.5 to 2 h on rotator at 4 °C. Then, the mix was washed three times in extraction buffer and analyzed by Western blotting using antibodies for NMIIB, GFP, or Borealin.

For the coimmunoprecipitation in the presence of AZD, 1–2 × 10^6^ HEK293T cells were seeded on a 60-mm dish. After attaching to the dish (10–24 h), cells were transfected with 6 μg DNA per plate, mixed with 36 μg L.PEI. Before harvesting, 100 nM AZD-1152 (Sigma-Aldrich) was added for 1 h. Cells were harvested with extraction buffer containing AZD. Cells were sonicated, and the coimmunoprecipitation assay was carried out as above.

### Direct pull-down assay

Ni^2+^-NTA beads were equilibrated in Buffer B containing 20 mM Tris-HCl pH 8, 275 mM NaCl, 5% glycerol, 1% NP-40, and 30 mM imidazole. His-survivin proteins (40–45 μg) were added to the beads in a final volume of 200 μl. The beads–survivin protein mix was incubated on a rotator at 4 °C for at least 40 min and washed twice with 300 μl Buffer B. Rod-B proteins (40–45 μg) were added to the beads–survivin complex in a final volume of 100 μl. For total protein input, 15 μl of the beads–proteins mix were added to 15 μl of SDS sample buffer. The bead–protein mix was incubated and washed as above and eluted with 30 μl buffer B containing 250 mM imidazole for at least 25 min. The beads were centrifuged for 5 min (>16,000*g*), and 25 μl of the supernatant was added to 25 μl SDS sample buffer. Proteins were analyzed on 10% to 12% SDS-PAGE gels and detected with Western blot using antibodies for Aurora-B and anti-NMIIB. Rod-B and survivin pull-down assay was performed as in (19).

### *In vitro* phosphorylation assay

Recombinant His-Aurora-B and Rod-B proteins were incubated in kinase buffer (20 mM Tris pH 7.5, 10 mM MgCl_2_, 1 mM DTT, 50 mM ATP). The protein mix was then incubated for 30 to 60 min at 30 °C. The reaction was stopped by adding SDS sample buffer to the protein mix. Kinase reactions were separated on SDS-PAGE and analyzed by Coomassie blue staining and Western blots using antibodies for Phospho-Threonine and quantified with ImageJ software.

For radioactive kinase assay, recombinant His-Aurora-B and with Rod-B proteins were incubated in kinase buffer (20 mM Tris pH 7.5, 10 mM MgCl_2_, 1 mM DTT, 50 mM cold ATP, and 1 μCi γ-32P-ATP). The protein mix was then incubated for 30 to 60 min at 30 °C. The reaction was stopped by adding SDS sample buffer to the protein mix. Kinase reactions were separated on SDS-PAGE. The SDS-PAGE gels were dried and imaged using a phosphoimager cassette (Molecular Dynamics) and a Typhoon Trio variable mode imager. Images from phosphoimager were quantified with ImageJ software.

### *In vivo* phosphorylation assay

A total of 3–4x10^6^ HEK293T cells were seeded on a 10-cm dish. After attaching to the dish (10–24 h), cells were cotransfected with 10 μg GFP-NMIIB constructs and 2.5 μg FLAG-Aurora-B mixed with 75 μg L.PEI. Cells were harvested 24 h post transfection with 500 μl NETN buffer (20 mM Tris-HCl pH 8.0, 100 mM NaCl, 1 mM EDTA, 0.5% NP-40, 50 mM β-glycerophosphate, and 100 mM NaF) supplemented with protease inhibitor cocktail (Sigma-Aldrich). Cell extracts were sonicated and centrifuged at 4 °C for at least 15 min (>16,000*g*). Anti-GFP antibodies were incubated with protein A/G beads (Santa Cruz Biotechnology) prewashed with 300 μl NETN buffer on a rotator for 1.5 to 2 h. The beads–antibodies mix was washed three times with NETN buffer and analyzed by Western blotting using antibodies for Phospho-Threonine, GFP, and FLAG.

The *in vivo* phosphorylation assay in the presence of AZD was carried out as above except that the cells were treated with dimethyl sulfoxide or 100 nM AZD for an hour before they were harvested.

### Immunofluorescence and live imaging

Cells were treated with 10 μM S-trityl-L-cysteine (STLC) for 12 to 16 h and harvested by mitotic shake-off, and 3–4 × 10^5^ cells were seeded on Poly-DL-Lysine (PDL)-coated coverslips. After attachment (∼10–15 min after seeding), the medium was replaced with fresh medium and cells were incubated for 2.5 h, fixed with 4% formaldehyde in PBS, washed three times with PBS, and permeabilized for 3 min with PBS containing 0.2% Triton X-100 and 0.5% BSA. After three washes with PBS, cells were blocked with horse serum diluted 1:50 for 35 min at 37 °C. Cells were washed, and primary antibodies in PBS containing 0.1% BSA were added and incubated for 2 h at 37 °C or overnight at 4 °C. Coverslips were washed three times with PBS, and secondary antibodies were added and incubated for 1 h at 37 °C. Where indicated, 300 nM DAPI (Sigma-Aldrich) was added to the coverslips and incubated for 5 min at room temperature. Coverslips were mounted on slides (Thermo Scientific) using Vectachield mounting medium (Vector Laboratories Inc).

For the Cos-7 rescue experiments, 50 to 100K cells were seeded on PDL-coated cover slips. After cells fully attached (about 16 h), they were treated with 0.2 mg/ml Doxycycline. Twenty-four hours post doxycycline treatment cells were transfected with 1.5 μg GFP-NMIIB constructs. Forty-eight hours post transfection cells were fixed and stained as described above.

For the 293T intercellular bridge assay, 300K cells were seeded on PDL-coated coverslip. After cells fully attached, they were transfected with 1.5 μg of GFP-NMIIB constructs. Twenty-four hours post transfection cells were synchronized using 10 μM STLC. About 12 to 16 h after synchronization cells were washed with PBS and released to a fresh medium. Three hours after release, cells were fixed and stained as described above. Confocal images were obtained with Nikon A1R with 1.4 CFI plan Apo Lambda 60 × oil objective or Nikon Yokogawa W1 Spinning Disk with CFI Plan-Apochromat Lambda 60× objective. Optical sections were collected at 500-nm interval. Image analyses and 3D reconstitution were carried out using NIS-Elements AR.

For live imaging, 1–2 × 10^6^ HeLa cells were seeded onto a 60-mm dish for 12 to 16 h, and then cells were transfected with 4.8 μg mCherry–NMIIB and 1.2 μg GFP-Aurora-B or 4.8 μg mCherry–Aurora-B and 1.2 μg GFP-NMIIB or 4.8 μg GFP-NMIIB constructs and 1.2 μg mCherry-tubulin. At 24 to 36 h post transfection, STLC was added for 12 to 16 h. Then 4 × 10^5^ cells were seeded in a PDL-coated chamber (Ibidi); after attachment, the medium was replaced with fresh medium and after 1 to 1.5 h, live cell imaging was carried out at 37 °C and 5% CO_2_. Live images were taken every 3 min using a Nikon Ti microscope with a Plan-Apochromat 63×/1.40 oil DIC M27 objective. When noted, 1 μg/ml Hoechst 33342 was used.

### Fluorescence quantification

Line scans of endogenous Aurora-B and NMIIB were generated along the division plane using the ImageJ software package (National Institutes of Health). For colocalization analysis, the PCC was calculated between the intensity profiles of NMIIB and Aurora-B along the cell cortex (for metaphase) and along the cleavage furrow (for anaphase and telophase) as described (19). The PCC was calculated using Excel (Microsoft) and Prism 6 (GraphPad). Statistical analysis was done using Prism 6. Data were examined by a two-tailed Student's *t* test. Normal cortical enrichment was determined by line scan drawn along the cell cortex. Cells that show two significant peaks of NMIIB were referred to as normal enriched cells.

### Rod-B solubility assay

The solubility assay was performed as described (62). Briefly, Rod-B (8.5 μg/120 μl) was dialyzed against Buffer G (10 mM phosphate buffer pH 7.5, 2 mM MgCl_2_, 1 mM DTT, and 200 mM NaCl) for 4 to 16 h at 4 °C. Then 100 μl of Rod-B was centrifuged at high speed (135,000*g*) using Beckman–Coulter centrifuge tubes. A volume of 80 μl from the supernatant (nonfilamentous NMII) was added to a fresh tube containing 20 μl 5 × SDS sample buffer. A volume of 100 μl Buffer G was added to the pellet (filamentous NMII), and the tubes were vortexed at 900 rpm for 30 min. Then 25 μl 5 × SDS sample buffer was added to the pellet. When indicated, 4 μg Aurora-B was added to Rod-B before dialysis. Samples were analyzed on 10% SDS-PAGE gels stained with Coomassie Brilliant Blue, scanned, and quantified using the densitometry program ImageJ. For the filament disassembly assay, recombinant His-Aurora-B was incubated in kinase buffer (20 mM Tris pH 7.5, 10 mM MgCl_2_, 1 mM DTT, and 50 μM cold ATP) with Rod-B proteins, and the protein mix was incubated for 60 min at 30 °C. Then, the protein mix was centrifuged and the supernatants and pellets were analyzed as described above.

### Electron microscopy

Electron microscopy was carried out as described (62). Briefly, purified Rod-B proteins in buffer G were diluted with buffer G to 0.5 mg/ml and then dialyzed against buffer Q (10 mM Tris-HCl pH 7.5, 50 mM NaCl, 20 mM MgCl_2_, 25 mM CaCl_2_) for 16 h at 4 °C in small dialysis tubes (miniGeBAflex tubes; catalog no. DO70-6-30). Five-microliter samples were placed on formvar/carbon-coated copper 200 mesh grids (EMS), mixed with 5 μl of 2% uranyl acetate for 5 to 10 s and the grids were dried. Grids were viewed with Jeol JEM-1400 Plus TEM (Jeol) equipped with ORIUS SC600 CCD camera (Gatan) and Gatan Microscopy Suite program (DigitalMicrograph, Gatan).

### Data availability

All data have been included within the article.

## Supporting information

This article contains supporting information (19).

## Conflict of interest

The authors declare that they have no conflicts of interest with the contents of this article.
