# Supplementary material for: Aurora-B phosphorylates the myosin II heavy chain to promote cytokinesis
Source: J Biol Chem. 2021 Jul 31;297(3):101024. doi: 10.1016/j.jbc.2021.101024 (PMC8385403; doi:10.1016/j.jbc.2021.101024)
Supplement: Figure S1–S4 [file mmc7.pdf]

Supplementary Information

Figure S1: Aurora-B and NMIIB colocalize during telophase. A. HeLa cells were seeded on PDL-coated coverslips, fixed, and immunostained for endogenous NMIIB (green) and Aurora-B (red). Shown are single Z-plane micrographs. Tubulin was used to determine the mitotic stage of each cell (not shown). Scale bar: 5 µm. **B**. Single Z-plane micrographs taken from a time-lapse movie of HeLa cells transiently expressing mCherry-NMIIB and GFP–Aurora B (Movie 1). Images were taken every 3 min. Scale bar: 10 µm.

Figure S2: Aurora-B inhibition causes mitotic defects. Single Z-plane micrographs taken from a time-lapse movie of HeLa cells transiently expressing GFP-NMIIB treated with AZD (Movie 4). Chromosomes were stained using Hoechst 33342. Images were taken every 3 min. Dashed lines represent the cell periphery. Scale bar: 10 µm.

Figure S3: Aurora-B phosphorylates Rod-B at T^{1847}. A. Left, purified Rod-B or Rod-B^{T1847A} were incubated with His-Aurora-B and subjected to phosphorylation assay in the presence of γ-32-ATP. Reaction was stopped using sample buffer. Proteins were loaded on SDS-PAGE and analyzed using phosphor-imager. Before the addition of γ-32P-ATP to the reaction mixture, half of the reaction was transferred to a fresh tube containing sample buffer for input. The input proteins were analyzed with SDS-PAGE and stained with coomassie brilliant blue. Right, phosphor-imager signals were normalized to the total amount of the protein loaded. Error bars show the SEM from three independent experiments. **B-C.** Position, residue, and score of possible Aurora-B phosphorylation sites that reside within NMIIB (B) and survivin (C). Score was obtained using the GPS software. Note that $survivin^{T117}$ was shown to be phosphorylated by Aurora B *in vitro*. **D.** Schematic presentation of NMIIB full length and Rod-B fragments. Amino acids 1827-1833 (marked in blue) are the survivin-binding domain (SBD) in NMIIB (1). T^{1847}, is the Aurora-B phosphorylation site (marked in red). This site is in close proximity to the SBD. ACD and cACD are assembly competent domain and complementary ACD, respectively. Rod-B and Rod-B^{288} represent the proteins used for the phosphorylation assay. **E.** Left, purified Rod-B^{288} or Rod-$B^{288\ T1847A}$ were incubated with His-Aurora-B and subjected to phosphorylation

assay in the presence of γ-32-ATP as in A. Right, phospho-imager signals were analyzed as described in A.

Figure S4: GFP-NMIIB^{T1847A} expression causes mitotic defects. A-B. Cos-7 cells were depleted for NMIIB and transfected with GFP-NMIIB (A) or GFP-NMIIB^{T1847A} (B). 48 hours post-transfection, cells were fixed and immunostained for GFP (green), tubulin (red), and DAPI (blue). Shown are single Z-plane micrographs. Scale bar: 10 µm. **C-D**. 293T cells were transfected with GFP-NMIIB (C) or GFP-NMIIB^{T1847A} (D). 48 hours after transfection, cells were fixed and immunostained for GFP (green), tubulin (red), and DAPI (blue). Shown are single Z-plane micrographs. Scale bar: 10 µm.

Movie 1 –HeLa cells transfected with GFP-Aurora-B and mCherry-NMIIB.

Movie 2 –HeLa cells transfected with GFP-NMIIB and treated with DMSO.

Movie 3 –HeLa cells transfected with GFP-NMIIB and treated with AZD.

Movie 4 – HeLa cells transfected with GFP-NMIIB and treated with AZD.

Movie 5 –HeLa cells transfected with GFP-NMIIB and mCherry-tubulin.

Movie 6 –HeLa cells transfected with GFP-NMIIB^{T1847A} and mCherry-tubulin.

References

1. Babkoff, A., Cohen-Kfir, E., Aharon, H., Ronen, D., Rosenberg, M., Wiener, R., and Ravid, S. (2019) A direct interaction between survivin and myosin II is required for cytokinesis. *J Cell Sci* **132**

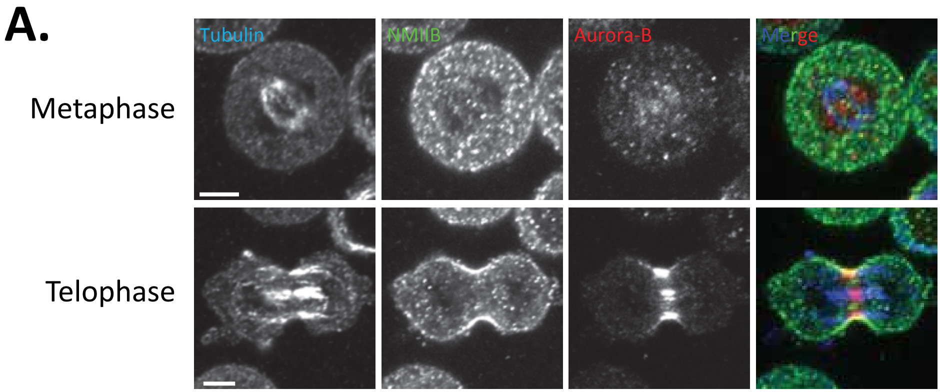

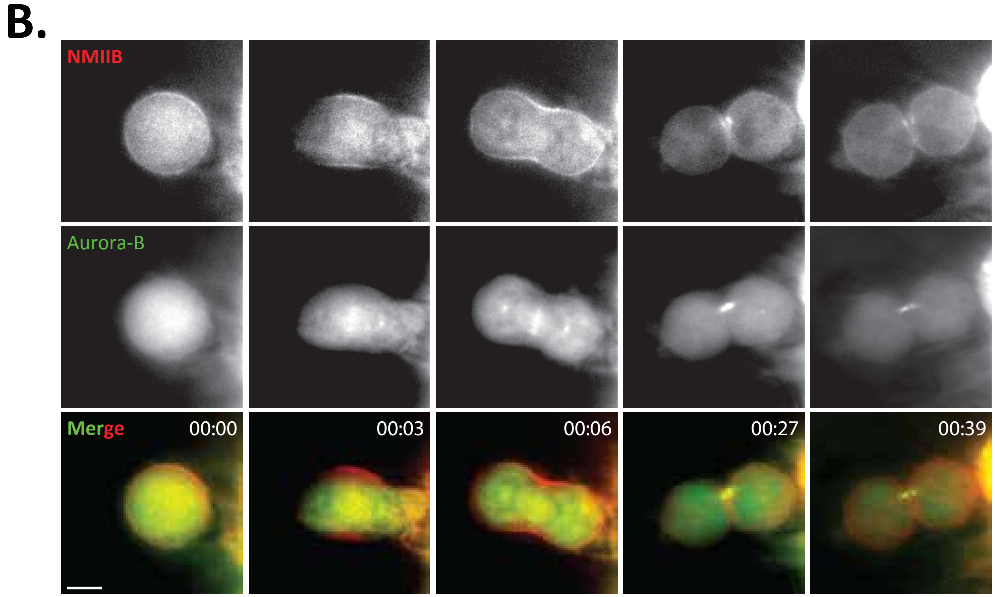

Figure S1: Aurora-B and NMIIB colocalize during telophase. A. HeLa cells were seeded on PDL-coated coverslips, fixed, and immunostained for endogenous NMIIB (green) and Aurora-B (red). Shown are single Z-plane micrographs. Tubulin was used to determine the mitotic stage of each cell (not shown). Scale bar: 5 µm. **B**. Single Z-plane micrographs taken from a time-lapse movie of HeLa cells transiently expressing mCherry-NMIIB and GFP–Aurora B (Movie 1). Images were taken every 3 min. Scale bar: 10 µm.

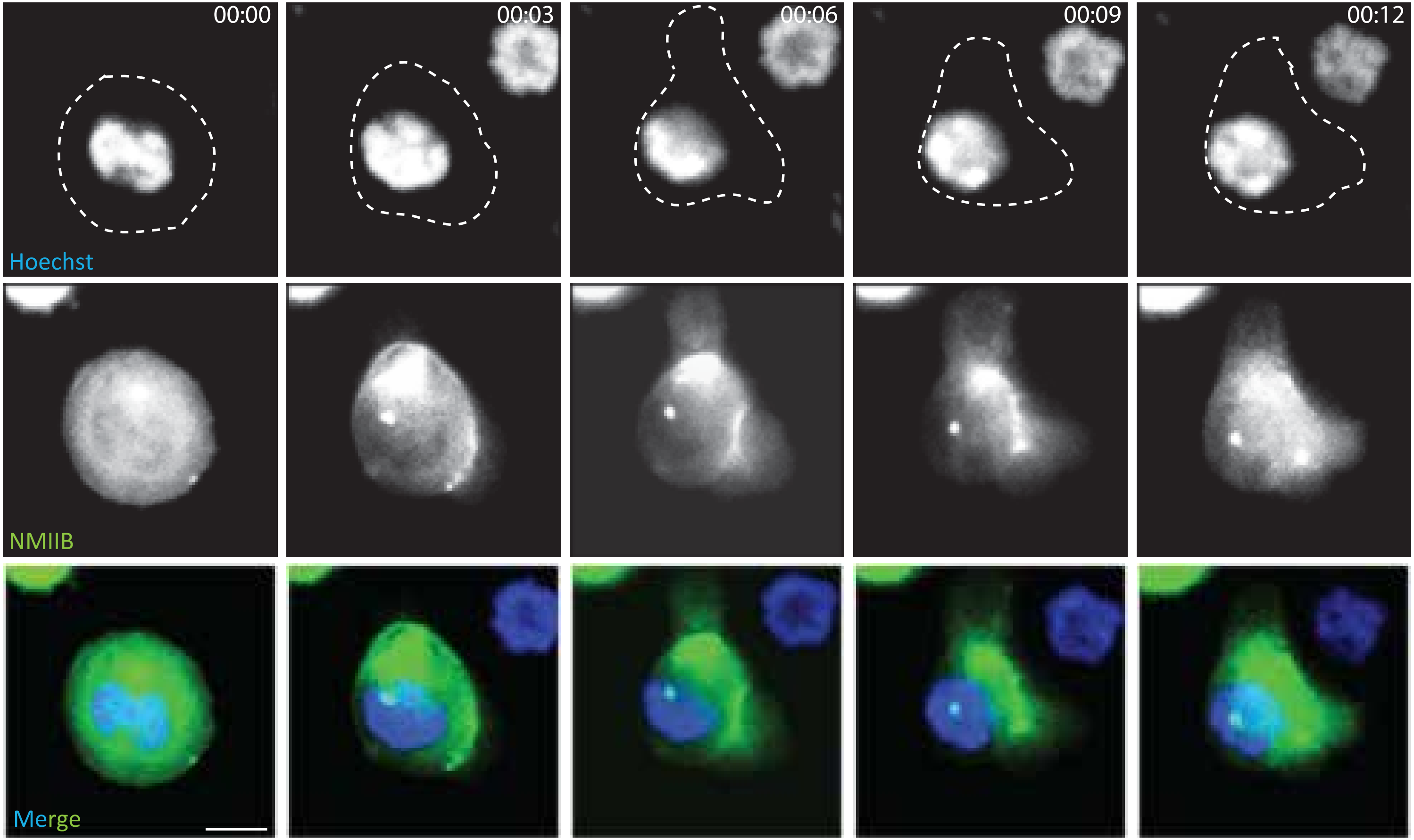

Fig. S2: Aurora-B inhibition causes mitotic defects. Single Z-plane micrographs taken from a time-lapse movie of HeLa cells transiently expressing GFP-NMIIB treated with AZD (Movie 4). Chromosomes were stained using Hoechst 33342. Dashed lines represent the cell periphery. Images were taken every 3 min. Scale bar: 10 µm.

A.

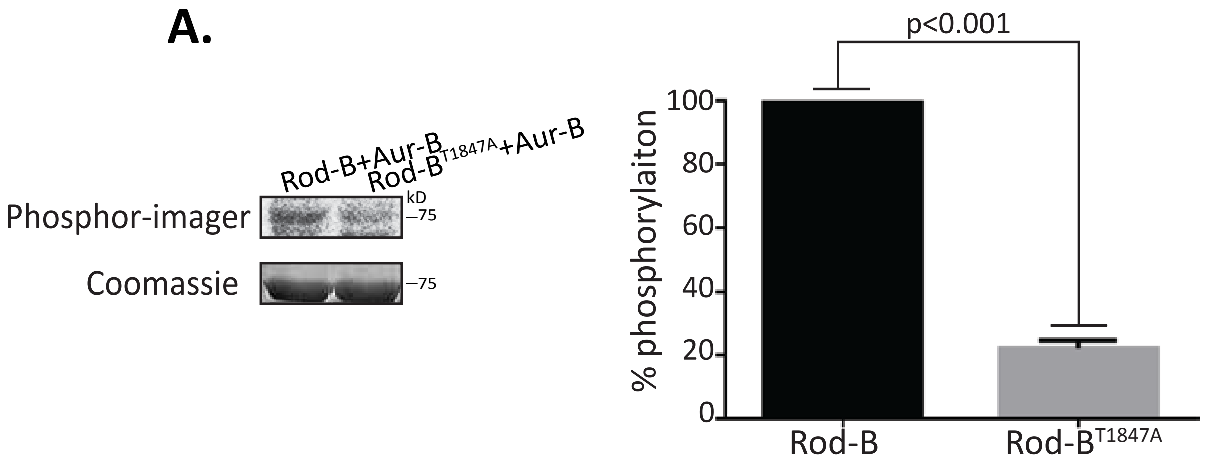

B.

NMIIB

	Position	Residue	Score
Head domain	367	Threonine	0.069
	671	Threonine	0.072
	787	Threonine	0.016
	1269	Serine	0.016
	1667	Serine	0.071
Tail domain	1762	Threonine	0.073
	1763	Threonine	0.017
	1847	Threonine	0.078

C.

survivin

Position	Residue	Score
20	Serine	0.073
81	Serine	0.012
117	Threonine	0.015

D.

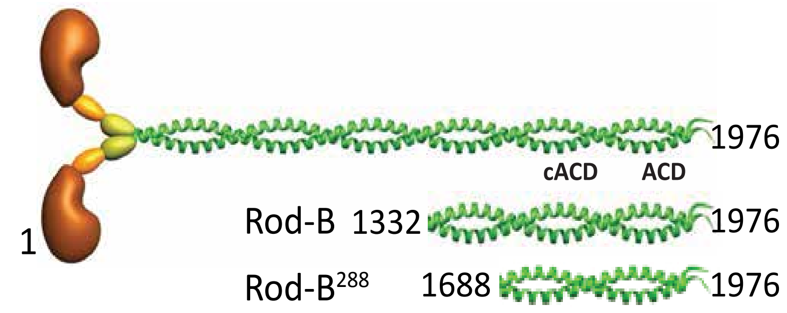

E.

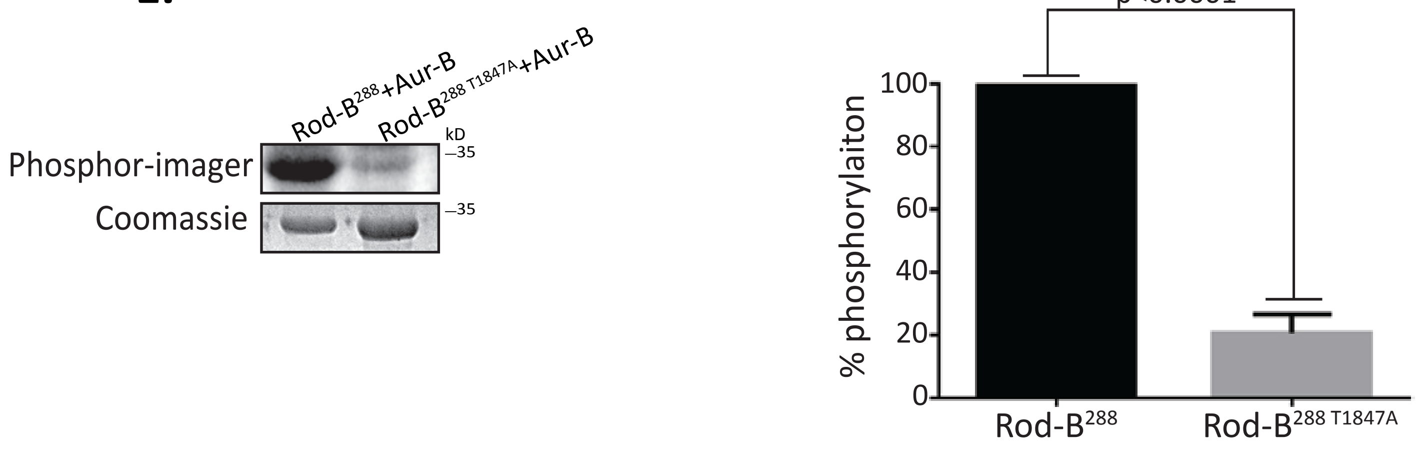

Figure S3: Aurora-B phosphorylates Rod-B at T^{1847}. A. Left, purified Rod-B or Rod-B^{T1847A} were incubated with His-Aurora-B and subjected to phosphorylation assay in the presence of γ-32-ATP. Reaction was stopped using sample buffer. Proteins were loaded on SDS-PAGE and analyzed using phosphor-imager. Before the addition of γ-32P-ATP to the reaction mixture, half of the reaction was transferred to a fresh tube containing sample buffer for input. The input proteins were analyzed with SDS-PAGE and stained with coomassie brilliant blue. Right, phosphor-imager signals were normalized to the total amount of the protein loaded. Error bars show the SEM from three independent experiments. **B-C.** Position, residue, and score of possible Aurora-B phosphorylation sites that reside within NMIIB (B) and survivin (C). Score was obtained using the GPS software. Note that $survivin^{T117}$ was shown to be phosphorylated by Aurora B *in vitro*. **D.** Schematic presentation of NMIIB full length and Rod-B fragments. Amino acids 1827-1833 (marked in blue) are the survivin-binding domain (SBD) in NMIIB (Babkoff et al., 2019). T^{1847}, is the Aurora-B phosphorylation site (marked in red). This site is in close proximity to the SBD. ACD and cACD are assembly competent domain and complementary ACD, respectively. Rod-B and Rod-B^{288} represent the proteins used for the phosphorylation assay. **E.** Left, purified Rod-B^{288} or Rod-$B^{288\ T1847A}$ were incubated with His-Aurora-B and subjected to phosphorylation assay in the presence of γ-32-ATP as in A. Right, phospho-imager signals were analyzed as described in A.

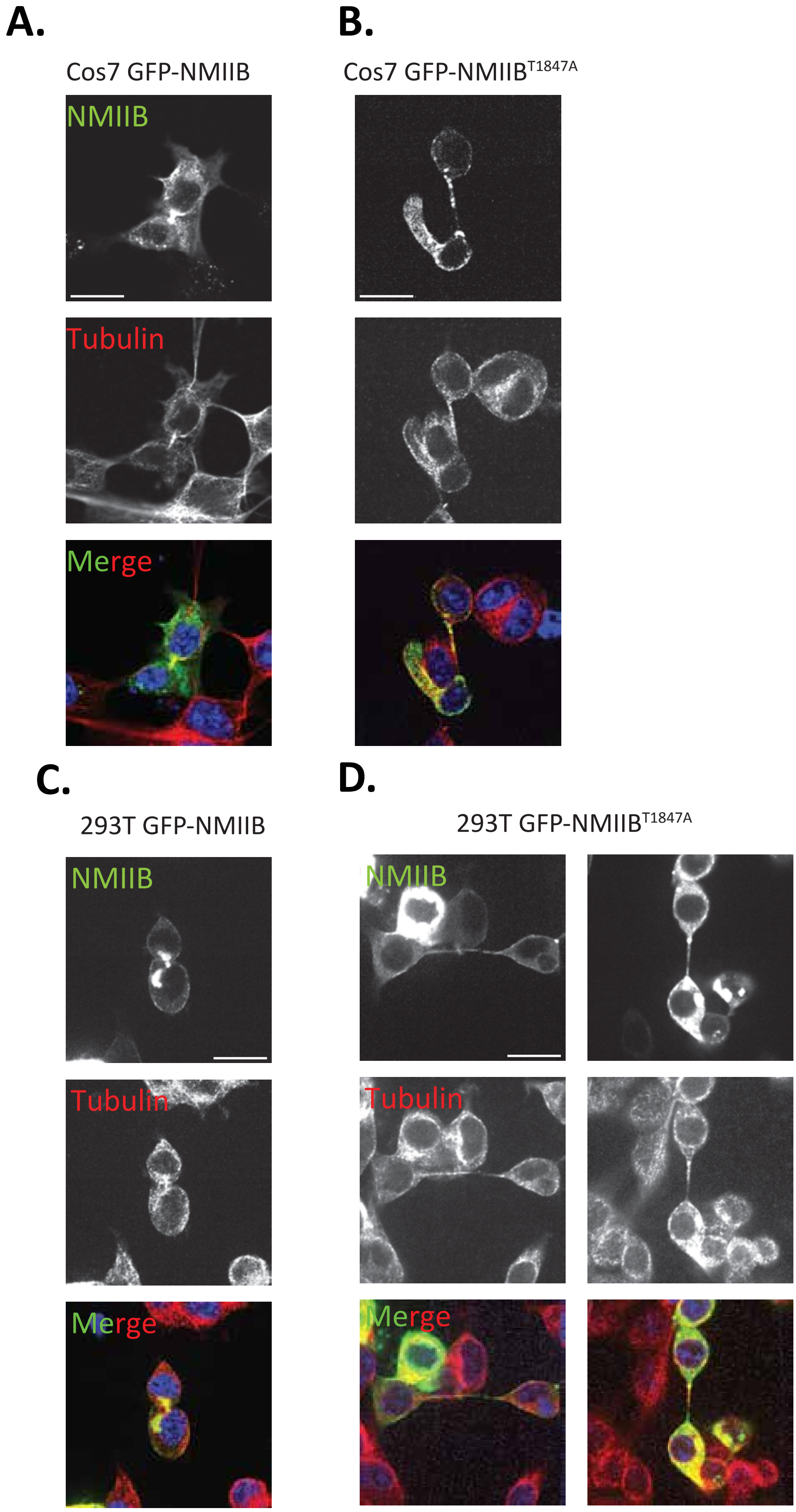

Figure S4: GFP-NMIIB^{T1847A} expression causes mitotic defects. A-B. Cos-7 cells were depleted for NMIIB and transfected with GFP-NMIIB (A) or GFP-NMIIB^{T1847A} (B). 48 hours post-transfection, cells were fixed and immunostained for GFP (green), tubulin (red), and DAPI (blue). Shown are single Z-plane micrographs. Scale bar: 10 µm. **C-D**. 293T cells were transfected with GFP-NMIIB (C) or GFP-NMIIB^{T1847A} (D). 48 hours after transfection, cells were fixed and immunostained for GFP (green), tubulin (red), and DAPI (blue). Shown are single Z-plane micrographs. Scale bar: 10 µm.
